# Deep neural networks and stochastic methods for cognitive modeling of rat behavioral dynamics in $\mathbb {T}$-mazes

**DOI:** 10.1007/s11571-025-10247-9

**Published:** 2025-04-25

**Authors:** Ali Turab, Josué-Antonio Nescolarde-Selva, Farhan Ullah, Andrés Montoyo, Cicik Alfiniyah, Wutiphol Sintunavarat, Doaa Rizk, Shujaat Ali Zaidi

**Affiliations:** 1 School of Software, Northwestern Polytechnical University, 127 West Youyi Road, Beilin District, Xi’an, 710072 China; 2 Department of Software and Computing Systems, University of Alicante, Alicante, Spain; 3 Department of Mathematics, Faculty of Science and Technology, Universitas Airlangga, 60115 Surabaya, Indonesia; 4 Department of Applied Mathematics, University of Alicante, Alicante, Spain; 5 Cybersecurity Center, Prince Mohammad Bin Fahd University, 617, Al Jawharah, Khobar, Dhahran 34754 Saudi Arabia; 6 Department of Mathematics and Statistics, Faculty of Science and Technology, Thammasat University Rangsit Center, 12120 Pathum Thani, Thailand; 7 Department of Mathematics, College of Science, Qassim University, 51452 Buraydah, Saudi Arabia; 8 Department of Computer Science, Faculty of Science, Chiang Mai University, Chiang Mai, Thailand

**Keywords:** Cognitive modeling, Animal decision-making, Spatial navigation, Analytical solution, Deep neural networks, 92D50, 47H10, 68T07, 9110

## Abstract

Modeling animal decision-making requires mathematical rigor and computational analysis to capture underlying cognitive mechanisms. This study presents a cognitive model for rat decision-making behavior in $\mathbb {T}$-mazes by combining stochastic methods with deep neural architectures. The model adapts Wyckoff’s stochastic framework, originally grounded in Bush’s discrimination learning theory, to describe probabilistic transitions between directional choices under reinforcement contingencies. The existence and uniqueness of solutions are demonstrated via fixed-point theorems, ensuring the formulation is well-posed. The asymptotic properties of the system are examined under boundary conditions to understand the convergence behavior of decision probabilities across trials. Empirical validation is performed using Monte Carlo simulations to compare expected trajectories with the model’s predictive output. The dataset comprises spatial trajectory recordings of rats navigating toward food rewards under controlled experimental protocols. Trajectories are preprocessed through statistical filtering, augmented to address data imbalance, and embedded using t-SNE to visualize separability across behavioral states. A hybrid convolutional-recurrent neural network (CNN-LSTM) is trained on these representations and achieves a classification accuracy of 82.24%, outperforming conventional machine learning models, including support vector machines and random forests. In addition to discrete choice prediction, the network reconstructs continuous paths, enabling full behavioral sequence modeling from partial observations. The integration of stochastic dynamics and deep learning develops a computational basis for analyzing spatial decision-making in animal behavior. The proposed approach contributes to computational models of cognition by linking observable behavior to internal processes in navigational tasks.

## Introduction

Mathematical models are essential tools for advancing scientific knowledge, especially once comprehensive descriptive statistics have been collected. These models help structure and interpret scientific findings while guiding future experimental research (see Tsibulsky and Norman 2007; An et al. 2024). In educational psychology, where data collection is abundant, the development of robust numerical models is highly feasible (see Tedeschi 2023; Clark 2018).

Research on spatial learning and decision-making in animals has long been a central focus in cognitive neuroscience and psychology (see Ma et al. 2023; Majhi et al. 2024). The $\mathbb {T}$-maze experiment is one of the most widely used frameworks for investigating these cognitive processes (see Ngoc Hieu et al. 2020; Gammeri et al. 2022). Despite its simple design, the $\mathbb {T}$-maze offers a sophisticated platform for exploring complex behaviors such as learning and memory (see Sharma et al. 2010; Wenk 1999).

In behavioral psychology, key concepts such as associative learning, categorization, reward, and inhibition are foundational for real-world applications. Despite a wealth of virtual studies on training and extinction, there remains a lack of critical research offering precise, quantifiable models to direct experimental work (see Couzin and Heins 2023; Babenko and Romanov 2024; Turab et al. 2022a). Decision-making research has yielded mixed results, particularly in light-guessing experiments and coalition games. While some models accurately predict behavior, others prompt further questions rather than solutions (see Turab et al. 2022b; Ofshe and Ofshe 1970). Theoretical learning experiments suggest that stochastic mechanisms, supported by occurrence theory, can be applied to basic learning studies in probability-learning contexts (see Estes and Straughan 1954; Grant et al. 1951). However, conflicting findings highlight the need for further research into functional equations that analyze animal behavior in two-choice scenarios (see Mosteller 2006; Turab and Sintunavarat 2019, 2020).

The study of animal behavior, particularly in rats, attracts interest from fields such as psychology, neuroscience, ethology, and pharmacology (see Luo et al. 2019; Li et al. 2022). The $\mathbb {T}$-maze test is one of the oldest and most trusted experimental setups for studying spatial cognition and behavior in rats (see Tolman 1948; O’keefe and Nadel 1979; Bush and Wilson 1956). While the maze may seem straightforward, it serves as an effective tool for investigating cognitive processes like memory retention and decision-making (see Oberto et al. 2023; d’Isa et al. 2021). On the other hand, Smith et al., in Smith et al. (2005), highlight a growing trend toward using mathematical models, rather than solely empirical or statistical methods, to study rats’ behavior in $\mathbb {T}$-mazes. Techniques such as Bayesian statistical theory and neural spike train analysis are increasingly employed to offer deeper insights into decision-making mechanisms (see Brown et al. 1998).

Examining how rats navigate spatial environments necessitates an interdisciplinary approach that bridges cognitive neuroscience and computational modeling. The hippocampal-entorhinal system plays a central role in this process, constructing an internal representation of space that facilitates memory integration and flexible learning. Recent theoretical advancements, such as the Tolman-Eichenbaum Machine (TEM), have provided a structured framework for understanding these cognitive processes. According to this model, medial entorhinal neurons generate abstract spatial representations, which hippocampal circuits then associate with sensory input, enabling adaptive decision-making across varied environments (see Whittington et al. 2020). This conceptualization aligns with empirical findings demonstrating that the hippocampus encodes spatial and relational knowledge through distinct neuronal firing patterns, including those of grid and place cells, allowing rodents to generalize learned information to novel contexts.

Advances in network science have deepened our understanding of the structural organization in biological systems by uncovering fundamental connectivity patterns that govern the emergence of complex morphological architectures (see Gosak et al. 2022). Transformer-based architectures, when modified with recurrent positional encoding, have been observed to spontaneously develop spatial representations resembling those found in the mammalian hippocampus, reinforcing the notion that neural-inspired deep learning models can approximate cognitive mapping mechanisms (Whittington et al. 2021). These insights contribute to a broader understanding of how the brain organizes and retrieves spatial information. In an effort to unify disparate perspectives on hippocampal function, recent studies have synthesized various models, illustrating how structural knowledge and past experiences interact to form coherent, flexible cognitive maps (see Whittington et al. 2022).

Beyond encoding spatial structures, the hippocampus also plays a predictive role in guiding behavior through dynamic processes such as replay. This mechanism allows neural circuits to reconstruct past experiences, rearranging familiar spatial elements to generate potential future pathways. Such recombination of stored information enables efficient decision-making, particularly in novel or uncertain scenarios, supporting the idea that hippocampal processing extends beyond mere memory storage to active inference and planning (for more detail, see Bakermans et al. (2023)).

On the other hand, fixed point methods are instrumental in establishing the existence and uniqueness of solutions across various complex mathematical problems. In the realm of differential equations, for instance, Banach’s fixed point theorem has been applied to demonstrate the existence and uniqueness of solutions to differential equations with boundary conditions by transforming the problem into a fixed-point equation in an appropriate function space (see Turab et al. 2024a, b; Hammad et al. 2021). Similarly, in the study of nonlinear integral equations, fixed point theorems have been utilized to prove the existence and uniqueness of solutions, as seen in recent research on multidimensional fixed-point theorems and their applications (Rashid et al. 2024; Sazaklioglu 2024). Moreover, in the context of functional equations within Banach algebras, the approximation of fixed points of the product of two operators has been explored to address complex equations. These examples underscore the versatility of fixed point methods in providing rigorous solutions to intricate problems across diverse mathematical disciplines (for more detail, see Combettes and Pesquet (2021); Turab and Sintunavarat (2023); Turab et al. (2023)).

This study aims to develop and assess a mathematical model that captures the complexity of rat behavior in a $\mathbb {T}$-maze environment. Our objective is to establish criteria for the existence and uniqueness of solutions to the functional equations that describe these behaviors using fixed-point methods. We will design and validate these mathematical models with data obtained from $\mathbb {T}$-maze experiments. Additionally, we utilize deep learning techniques, such as Convolutional Neural Networks (CNNs) combined with Long Short-Term Memory (LSTM) units and Gated Recurrent Units (GRUs), to analyze rat navigation patterns. These advanced methods provide a comprehensive understanding of rats’ decision-making processes within the maze.

This research is significant because it advances scientific and mathematical knowledge of rat behavior in $\mathbb {T}$-mazes. The models we develop will enhance experimental design, data interpretation, and the development of computational methods for behavioral analysis. Furthermore, these mathematical frameworks could have broader applications, serving as foundational tools for research in behavioral psychology and neuroscience.

The key contributions of this study include:

- Demonstrating the effectiveness of fixed-point methods in creating a solid mathematical framework for predicting rat behavior in $\mathbb {T}$-maze experiments.
- Establishing clear criteria for the existence and uniqueness of solutions to specific stochastic functional equations.
- Validating the mathematical models through alignment with empirical data obtained from $\mathbb {T}$-maze experiments, confirming accuracy and practical relevance.
- Highlighting the potential of mathematical models to improve scientific understanding and solve real-world challenges in the analysis of animal behavior.

This paper delves into the intricate field of behavioral science, specifically examining rats’ cognitive and decision-making processes within the $\mathbb {T}$-maze framework. It begins with an introduction that outlines the study’s background, objectives, and significance. This is followed by a comprehensive literature review detailing prior research on $\mathbb {T}$-mazes, the role of mathematical modeling in behavioral studies, and the application of probability and learning theory. The methodology section explains the $\mathbb {T}$-maze setup and the experimental design. A thorough mathematical analysis is presented, incorporating advanced techniques like fixed-point theory and behavior modeling. The study then validates the proposed models using deep learning methods and conducts an in-depth analysis of the results. Finally, the paper summarizes the main findings and identifies potential areas for future research in this rapidly evolving field.

## Literature review

The study of rat behavior in $\mathbb {T}$-maze experiments has been instrumental in advancing our understanding of cognitive processes, spatial learning, and decision-making. Early research by Tolman introduced the concept of cognitive maps, suggesting that rats develop internal representations of their environment rather than relying solely on stimulus–response associations (Tolman 1948). This foundational work laid the groundwork for neuroscientific explorations, leading to the discovery of place cells in the hippocampus by O’Keefe and Nadel, which provided direct neurophysiological evidence of spatial encoding (O’keefe and Nadel 1979; Bush and Wilson 1956). Over time, the $\mathbb {T}$-maze has remained a key apparatus for studying memory retention, reward-based learning, and the influence of pharmacological agents on cognitive function (Wang and Salmaniw 2023; Danieli et al. 2023). Its versatility has enabled researchers to implement modifications such as delayed alternation tasks and spatial navigation challenges, further broadening its applicability in behavioral research (see Knowlton and Castel 2022).

Mathematical modeling has emerged as a crucial tool for formalizing hypotheses and generating quantitative predictions in animal behavior studies. The integration of stochastic models has allowed researchers to capture the probabilistic nature of decision-making, particularly in structured experimental settings like $\mathbb {T}$-mazes (Smith et al. 2005; Tuqan and Porfiri 2021). These models, often formulated using Markov decision processes and stochastic differential equations, provide insights into how behavioral choices evolve over time (see Wijeyakulasuriya et al. 2020; Ghanbari and Djilali 2020). More recently, fixed-point methods have been applied to establish the existence and uniqueness of solutions in these models, strengthening the computational foundations of behavioral analysis (Luxem et al. 2022; Shi et al. 2020). The intersection of probability theory and learning mechanisms further supports this approach, aligning with Bayesian inference models that describe how prior knowledge is updated based on new observations (Conway 2020). In this context, neural computation and synaptic plasticity have been shown to reflect statistical regularities in the environment, consistent with Hebbian learning principles (Mazzucato 2022).

The reinforcement learning paradigm has also been instrumental in understanding decision-making processes in both artificial and biological systems (see Hao et al. 2025). Inspired by behavioral psychology, models such as the Rescorla-Wagner framework and temporal difference learning algorithms capture the principles of adaptive learning through reward-driven mechanisms (Ernst and Louette 2024; Navarro et al. 2024). Neuropsychological theories, including the free-energy principle, further emphasize how organisms minimize uncertainty and prediction errors by dynamically updating their internal models (Sánchez-Cañizares 2021; Cook et al. 2022). These approaches have provided a unified perspective on learning and cognition, bridging the gap between theoretical modeling and empirical observations (see Bai et al. 2024).

Advancements in computational methods have further enhanced our ability to analyze complex behavioral patterns (see Gao et al. 2021). The integration of deep learning with traditional stochastic models has enabled the study of hierarchical structures in large-scale animal movement data, improving behavioral classification and predictive accuracy (Dehghani and Trojovský 2022; Torney et al. 2021). In particular, neural networks have demonstrated remarkable efficacy in capturing nonlinear dependencies in behavioral datasets, contributing to more explainable and interpretable models of decision-making (Goodwin et al. 2022). The synergy between machine learning and mathematical modeling has resulted in hybrid approaches that provide a comprehensive framework for decoding animal cognition, particularly in controlled experimental environments such as $\mathbb {T}$-mazes (Turab et al. 2024). Recent studies have further emphasized the role of neural mechanisms in structuring naturalistic animal behavior, reinforcing the importance of computational techniques in behavioral ecology and cognitive neuroscience (see Mazzucato (2022)).

## Methodology

### Setting of $\mathbb {T}$-maze for spatial learning

**Fig. 1:**
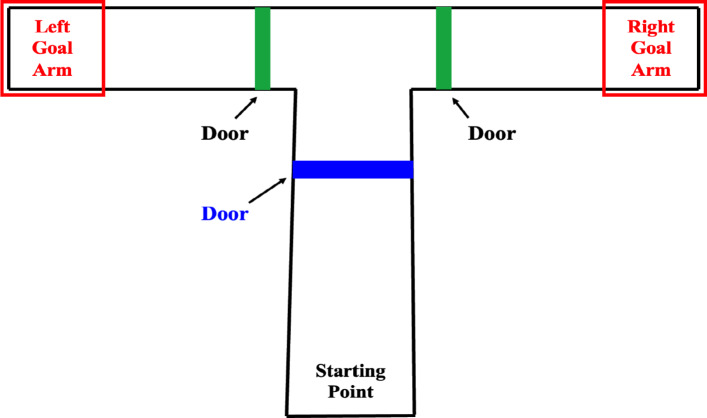
Overview of the $\mathbb {T}$-maze apparatus showing the starting point, goal arms, and door placements

The $\mathbb {T}$-maze is a crucial experimental tool commonly used in neuroscience and psychology to study spatial learning and decision-making in animals, particularly rodents such as mice and rats. This maze consists of a long, linear corridor called the “stem”, which ends at a junction resembling the shape of a “$\mathbb {T}$”. The junction branches into two arms positioned at right angles to the stem, offering the animal a choice between two distinct paths, typically referred to as the “left” and “right” arms (see Fig. 1 below).

**Fig. 2:**
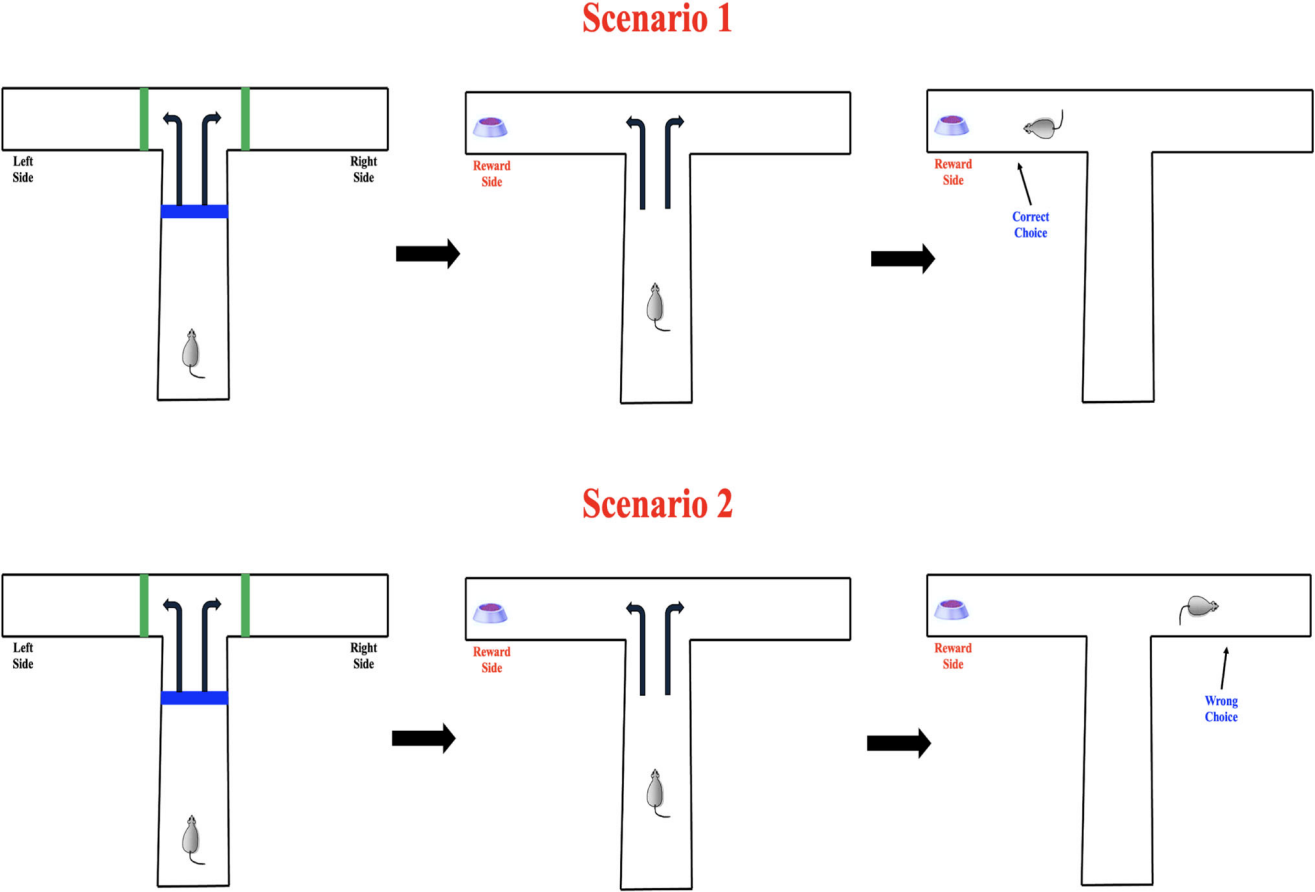
Two decision-making outcomes in the $\mathbb {T}$-maze task. In Scenario 1 (top), the rat selects the reward side correctly. In Scenario 2 (bottom), the rat chooses the non-rewarded side, reflecting an incorrect decision under identical conditions

Researchers use the $\mathbb {T}$-maze to investigate a variety of cognitive and behavioral processes, including memory retention, decision-making patterns, and the effects of pharmacological interventions. The maze’s design can be modified with rewards, barriers, or cues, allowing for a wide range of experimental setups. Its simplicity and adaptability make the $\mathbb {T}$-maze a popular tool in behavioral research (see Fig. 2 below).

### Experimental design

**Fig. 3:**
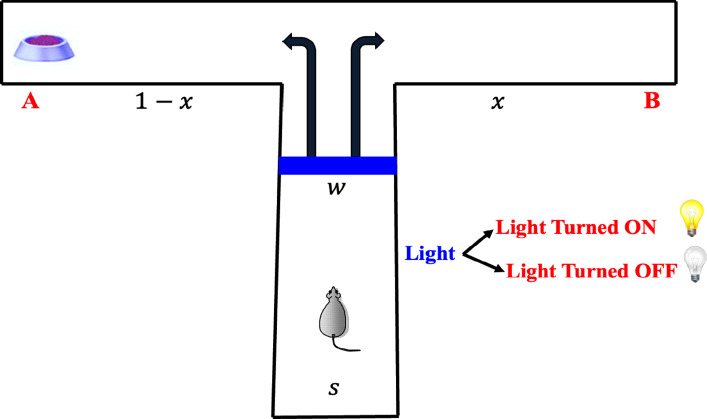
Illustration of a rat’s behavior in a $\mathbb {T}$-maze task, where a light cue determines the probabilistic preference toward goal arm *A* or *B*. The rat starts from point *S*, passes through the central region *w*, and makes a directional choice based on the presence or absence of the light cue

Bush’s linearization of Wyckoff’s theory provides the foundation for the model being presented (see Luce et al. 1963; Bush 1959). The experimental setup includes two lights, **L** and $\tilde{\textbf{L}}$, located at the choice point (see Fig. 3). A rat starts at the initial position $\textbf{s}$ and moves to a decision point, $\textbf{w}$. From there, it selects one of two containers, $\textbf{A}$ or $\textbf{B}$, where food may be available. On each trial, one of the lights is randomly lit, with food offered either on the left (**A**) or right (**B**), depending on whether **L** or $\tilde{\textbf{L}}$ is illuminated-each occurring randomly in 50% of the trials. Over time, most rats will learn to make the correct choice consistently during such experiments.

Let $x_{n}$ represent the probability that the rat notices which light is on during trial *n* (event $\tilde{{\textbf {T}}_{n}}$). Let $u_{1,n}$ and $u_{2,n}$ denote the probabilities of obtaining food (event ${\textbf {O}}_{n}$) on **L** and $\tilde{\textbf{L}}$ trials, assuming the rat is attentive to the lights. If the rat ignores the lights, the probability of receiving food is $\frac{1}{2}$. The likelihood $u_{i}$ of getting food increases if a rewarded response follows attention, or if a nonrewarded response follows inattention. These are the only conditions under which $u_{i}$ changes. The probability *x* of attending to the lights increases if attention is followed by reward, or if inattention is followed by nonreward. Conversely, it decreases in opposite cases. All changes are assumed to occur through linear functions.

**Fig. 4:**
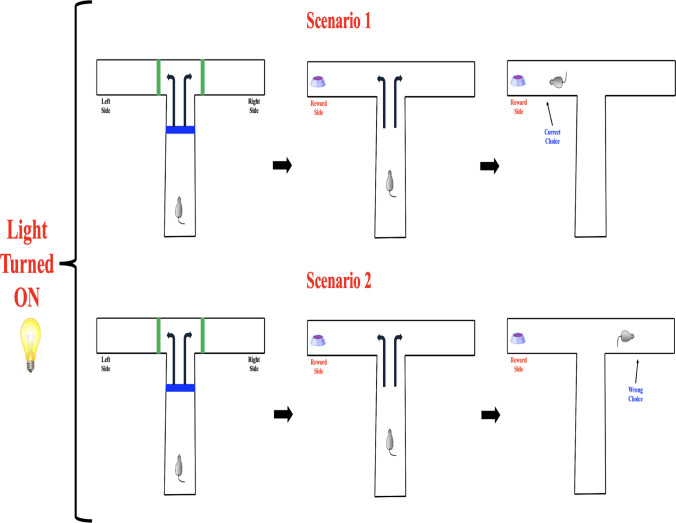
Possible response outcomes in the $\mathbb {T}$-maze task when a light cue is presented. In both scenarios, the rat is exposed to an illuminated cue signaling the availability of reward. Scenario 1 (top) illustrates a correct choice toward the reward side, while Scenario 2 (bottom) shows an incorrect decision despite identical cue conditions

**Fig. 5:**
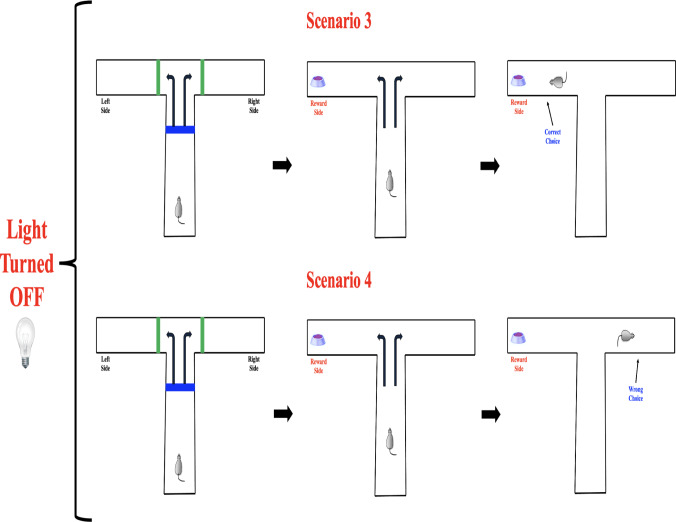
Possible response outcomes in the $\mathbb {T}$-maze task when the light cue is absent. Scenario 3 (top) shows a correct choice toward the reward side without an external cue. Scenario 4 (bottom) illustrates an incorrect choice made under the same cue-absent condition, indicating behavioral variability without stimulus guidance

**Fig. 6:**
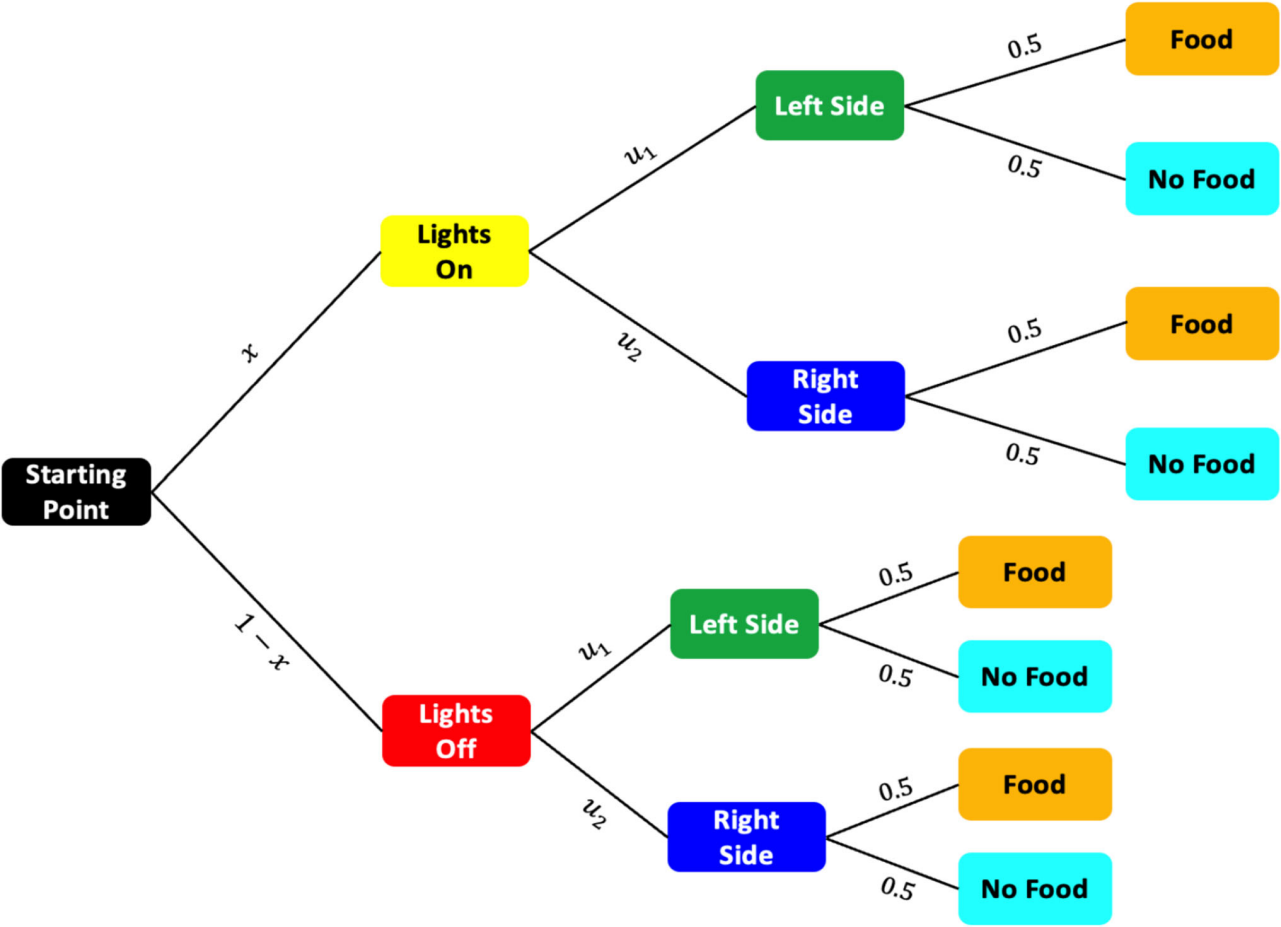
Probability tree diagram representing possible outcomes in the $\mathbb {T}$-maze task. Starting from the initial point, the rat encounters a light cue with probability *x* or no cue with probability $1 - x$. In each condition, it chooses between the left and right arms with probabilities $u_1$ and $u_2$, respectively. Each directional choice results in a reward or no reward with equal probability

These response patterns are systematically represented using a probability tree diagram, a graphical structure that illustrates sequential decision-making processes. In this model, each branching point represents a decision or event, with branches corresponding to possible outcomes and their associated probabilities. The probability tree diagram (see Figs. 4, 5 and 6 below) provides a structured visualization of how response probabilities evolve based on prior experiences.

The summary of these assumptions is provided in Table 1.

**Table 1:** Events and their interpretations occurred in the $\mathbb {T}$-maze test

Event	Choosing side	Outcome	Probability
$\mathbf {LT:}$ Light on	$\mathbf {A:}$ Left side	$\textbf{O}_{1}\mathbf {:}$ Food side (reward)	$\frac{1}{2}u_{1}x$
${\textbf{L}}{\textbf{T}}:$ Light on	$\mathbf {A:}$ Left side	$\mathbf {O_{2}:}$ Non-food side (no reward)	$\frac{1}{2}(1-u_{1})x$
$\tilde{\textbf{L}T}:$ Light on	$\mathbf {B:}$ Right side	$\mathbf {O_{1}:}$ Food side (reward)	$\frac{1}{2}u_{2}x$
$\tilde{\textbf{L}T:}$ Light on	$\mathbf {B:}$ Right side	$\textbf{O}_{2}\mathbf {:}$ Non-food side (no reward)	$\frac{1}{2}(1-u_{2})x$
$\tilde{\textbf{T}:}$ Both lights off	$\mathbf {A:}$ Left side	$\mathbf {O_{1}:}$ Food side (reward)	$\frac{(1-x)u_{1}}{2}$
$\tilde{\textbf{T}:}$ Both lights off	$\mathbf {A:}$ Left side	$\mathbf {O_{2}:}$ Non-food side (no reward)	$\frac{(1-x) (1-u_{1})}{2}$
$\tilde{\textbf{T}:}$ Both lights off	$\mathbf {B:}$ Right side	$\mathbf {O_{1}:}$ Food side (reward)	$\frac{(1-x)u_{2}}{2}$
$\tilde{\textbf{T}:}$ Both lights off	$\mathbf {B:}$ Right side	$\mathbf {O_{2}:}$ Non-food side (no reward)	$\frac{(1-x)(1-u_{2})}{2}$

The chain of actions presented above describes a single experimental test. This procedure is typically repeated multiple times with the same rat. The overall activity of the rat in a maze experiment is complex. When the rat reaches the choice point, it is in one of two stimulus conditions. These conditions can be categorized, and specific metrics can be used to analyze various aspects of the rat’s behavior. For example, one might measure the latency from the starting point $\textbf{s}$ or analyze the momentum between $\textbf{s}$ and $\textbf{w}$. However, the rat’s decision at the choice point $\textbf{w}$ is the most critical aspect of this experiment.

In the following research, we focus solely on the rat’s route choice during each trial rather than analyzing other behaviors. Repeated experimental trials, with consistent stimuli, lead the rat to make a decision at the choice point. In Table 1, the $\mathbb {T}$-maze presents two options, each corresponding to one of the two possible target boxes, $\textbf{A}$ or $\textbf{B}$. Only one of these options is selected in each trial. In contrast to an individual trial, experimental research allows the rat to choose between two mutually exclusive and exhaustive alternatives.

## Theoretical framework

### Mathematical foundation and assumptions

The following fixed point outcome is required in the progression (to obtain further information regarding fixed point theory, one may refer (Jiang 2022; Hazarika et al. 2024)).

#### Theorem 4.1

(Banach 1922) Let $(\mathcal {A},d )$ be a complete metric space and $\mathcal {W}:\mathcal {A}\rightarrow \mathcal {A}$ be a Banach contraction mapping (shortly as, BCM) given by

4.1 $$\begin{aligned} d \left( \mathcal {W}\ell _{1},\mathcal {W}\ell _{2}\right) \le \wp d \left( \ell _{1},\ell _{2} \right) , \quad \forall \ell _{1},\ell _{2}\in \mathcal {A}, \end{aligned}$$

for $\wp <1$. Then $\mathcal {A}$ has one fixed point. In addition, the iteration $\{\ell _{1}^{(n)}\}$ in $\mathcal {A}$ defined as $\ell _{1}^{(n)}=\mathcal {W}\ell _{1}^{(n-1)}$ for all $n\in \mathbb {N}$, where $\ell _{1}^{(0)}\in \mathcal {A}$, converges to the unique fixed point of $\mathcal {W}$.

For the computational convenience, we use the following notations:

4.2 $$\begin{aligned} \mathfrak {K}_{1}^{\star }= & \left| \begin{array}{c} u_{1}\left( \vartheta _{1} + \vartheta _{5} + \frac{1}{2} (1 - \vartheta _{1}) \lambda _{1} + \frac{1}{2} (1-\vartheta _{5})\lambda _{5}) \right) \\ + (1 - u_{1}) \left( \vartheta _{2} + \vartheta _{6} + \frac{1}{2} (1 - \vartheta _{2}) \lambda _{2} + \frac{1}{2} (1-\vartheta _{6})\lambda _{6}) \right) \\ + u_{2}\left( \vartheta _{3} + \vartheta _{7} + \frac{1}{2} (1 - \vartheta _{3}) \lambda _{3} + \frac{1}{2} (1-\vartheta _{7})\lambda _{7}) \right) \\ + (1 - u_{2}) \left( \vartheta _{4} + \vartheta _{8} + \frac{1}{2} (1 - \vartheta _{4}) \lambda _{4} + \frac{1}{2} (1-\vartheta _{8})\lambda _{8}) \right) \end{array} \right| . \end{aligned}$$

4.3 $$\begin{aligned} \mathfrak {K}_{2}^{\star }= & \left| \begin{array}{c} u_{1} \left( \lambda + \left( 1 - \frac{\lambda }{2} \right) (\vartheta _{1} + \vartheta _{5}) \right) + (1 - u_{1} ) \left( \lambda + \left( 1 - \frac{\lambda }{2} \right) (\vartheta _{2} + \vartheta _{6}) \right) \\ u_{2} \left( \lambda + \left( 1 - \frac{\lambda }{2} \right) (\vartheta _{3} + \vartheta _{3}) \right) + (1 - u_{2} ) \left( \lambda + \left( 1 - \frac{\lambda }{2} \right) (\vartheta _{4} + \vartheta _{8}) \right) \end{array} \right| . \end{aligned}$$

4.4 $$\begin{aligned} \mathfrak {K}_{3}^{\star }= & \left| \begin{array}{c} u_{1}(\vartheta _{1} + \vartheta _{5}) + (1 - u_{1} ) (\vartheta _{2} + \vartheta _{6}) \\ + u_{2}(\vartheta _{3} + \vartheta _{7}) + (1 - u_{2} ) (\vartheta _{4} + \vartheta _{8}) \end{array} \right| . \end{aligned}$$

4.5 $$\begin{aligned} \mathfrak {K}_{4}^{\star }= & \left| \begin{array}{c} u_{1}( 1 + \vartheta _{1} + \vartheta _{5}) + (1 - u_{1}) ( 1 + \vartheta _{2} + \vartheta _{6}) \\ + u_{2}( 1 + \vartheta _{3} + \vartheta _{7}) + (1 - u_{2}) ( 1 + \vartheta _{4} + \vartheta _{8}) \end{array} \right| . \end{aligned}$$

### Model formulation

In this experiment, both the movement of the rat towards a particular compartment ($\textbf{A}$ or $\textbf{B}$) and the location of the food serve as critical cues. The rat’s behavior is monitored to see whether it moves to the left or right of where the food is placed. If the rat chooses the food side, event $\mathbf {O_{1}}$ occurs; otherwise, event $\mathbf {O_{2}}$ arises when the rat moves to the opposite side. Based on the rat’s movement, the light conditions (${\textbf {L}}$ and $\tilde{\textbf{L}}$), and the location of the food, there are eight possible outcomes, as shown in Table 2.

**Table 2:** The rat’s movement and the eight probable events

Response	Choosing side	Outcome	Event
$\textbf{LT}$	$\textbf{A}$	$\mathbf {O_{1}}$	$\mathbf {E_{1}}$
$\textbf{LT}$	$\textbf{A}$	$\mathbf {O_{2}}$	$\mathbf {E_{2}}$
$\tilde{\textbf{L}T}$	$\textbf{B}$	$\mathbf {O_{1}}$	$\mathbf {E_{3}}$
$\tilde{\textbf{L}T}$	$\textbf{B}$	$\mathbf {O_{2}}$	$\mathbf {E_{4}}$
$\tilde{\textbf{T}}$	$\textbf{A}$	$\mathbf {O_{1}}$	$\mathbf {E_{5}}$
$\tilde{\textbf{T}}$	$\textbf{A}$	$\mathbf {O_{2}}$	$\mathbf {E_{6}}$
$\tilde{\textbf{T}}$	$\textbf{B}$	$\mathbf {O_{1}}$	$\mathbf {E_{7}}$
$\tilde{\textbf{T}}$	$\textbf{B}$	$\mathbf {O_{2}}$	$\mathbf {E_{8}}$

The responses $\textbf{T}$ and $\tilde{\textbf{T}}$ occur with probabilities *x* and $(1-x)$, respectively. The experimental design assigns probabilities to the outcomes, determining whether the rat receives food in the presence of lights $\textbf{L}$ or $\tilde{\textbf{L}}$, with the probabilities $u_{1}$ and $u_{2}$, where $u_{1}, u_{2} \in [0,1]$. Table 3 presents the probabilities for each of the eight possible outcomes.

**Table 3:** Probability corresponding to each of the eight outcomes

Event	Probability of incident
$\mathbf {E_{1}}$	$\frac{1}{2}u_{1}x$
$\mathbf {E_{2}}$	$\frac{1}{2}(1-u_{1})x$
$\mathbf {E_{3}}$	$\frac{1}{2}u_{2}x$
$\mathbf {E_{4}}$	$\frac{1}{2}(1-u_{2})x$
$\mathbf {E_{5}}$	$\frac{(1-x)u_{1}}{2}$
$\mathbf {E_{6}}$	$\frac{(1-x)(1- u_{1})}{2}$
$\mathbf {E_{7}}$	$\frac{(1-x)u_{2}}{2}$
$\mathbf {E_{8}}$	$\frac{(1-x)(1- u_{2})}{2}$

Let $\vartheta _{1},\vartheta _{2}, \ldots , \vartheta _{8}\in (0,1)$ represent learning-rate parameters, which reflect the effect of the outcomes $\mathbf {E_{1}}-\mathbf {E_{8}}$ on modifying response probabilities. Additionally, let $\lambda _{k}\in [0,1]$, where $k=1,2,...,8$, be the fixed point of the associated events. For instance, if $\frac{1}{2}u_{1}x$ represents the probability of a response $\textbf{LT}$ with an outcome $\mathbf {O_{1}}$, and $\textbf{A}$ occurs, then the updated probability of $\textbf{LT}$ resulting in $\mathbf {O_{1}}$ will be $\vartheta _{1}x+(1-\vartheta _{1})\lambda _{1}$. Similarly, for a response $\textbf{LT}$ with outcome $\mathbf {O_{2}}$, the new probability becomes $\vartheta _{2}x+(1-\vartheta _{2})\lambda _{2}$.

To formalize these probability updates, we introduce the transition operators $P_{1}, P_{2}, \ldots , P_{8}$, which are mathematical mappings from $[0,1] \rightarrow [0,1]$. These operators define how response probabilities evolve over time-based on past events, ensuring that the decision-making process remains consistent with the model’s stochastic framework. The transition operators are defined as follows:

4.6 $$\begin{aligned} \left\{ \begin{aligned} P_{1}x&=\vartheta _{1}x+(1-\vartheta _{1})\lambda _{1},\\ P_{2}x&=\vartheta _{2}x+(1-\vartheta _{2})\lambda _{2}, \\ P_{3}x&=\vartheta _{3}x+(1-\vartheta _{3})\lambda _{3}, \\ P_{4}x&=\vartheta _{4}x+(1-\vartheta _{4})\lambda _{4}, \\ P_{5}x&=\vartheta _{5}x+(1-\vartheta _{5})\lambda _{5}, \\ P_{6}x&=\vartheta _{6}x+(1-\vartheta _{6})\lambda _{6} \\ P_{7}x&=\vartheta _{7}x+(1-\vartheta _{7})\lambda _{7} \\ P_{8}x&=\vartheta _{8}x+(1-\vartheta _{8})\lambda _{8} \end{aligned}\right. \end{aligned}$$

for all $$x\in [0,1].$$

These transition probabilities reflect the weighted sum of *x* and $\lambda _k$, with the learning rate parameters ensuring adaptation in response to the outcomes. If $\vartheta _{1}, \vartheta _{2}, \ldots , \vartheta _{8}$ all equal 1, the system would stabilize, ceasing further change in response probabilities. Given *x*, $\vartheta _{1},\vartheta _{2},\ldots ,\vartheta _{8}$, and $\lambda _{1},\lambda _{2},\ldots ,\lambda _{8}$, the probability of the rat ceasing to respond to one choice while consistently responding to the other can be expressed as:

$$\begin{aligned} P(x, \vartheta _{1}, \ldots , \vartheta _{8}, \lambda _{1}, \ldots , \lambda _{8}). \end{aligned}$$

This equation accounts for the response to various cues such as the pathway, light, and outcome, updating the probabilities according to the transitions described in (4.6). The final probability after each trial is computed by aggregating the event probabilities and the parameters $\vartheta _{i}$ and $\lambda _{i}$ for each event, as shown below:

4.7 $$\begin{aligned} P(x,\vartheta _{i},\lambda _{i})= & \frac{1}{2}u_{1} xP(\vartheta _{1}x+(1-\vartheta _{1})\lambda _{1},\vartheta _{i},\lambda _{i}) \nonumber \\ & +\frac{1}{2}(1-u_{1}) x P(\vartheta _{2}x+(1-\vartheta _{2})\lambda _{2},\vartheta _{i},\lambda _{i}) \nonumber \\ & +\frac{1}{2}u_{2} x P(\vartheta _{3}x+(1-\vartheta _{3})\lambda _{3},\vartheta _{i},\lambda _{i})\nonumber \\ & +\frac{1}{2}(1-u_{2}) xP(\vartheta _{4}x+(1-\vartheta _{4})\lambda _{4},\vartheta _{i},\lambda _{i}) \nonumber \\ & + \frac{1}{2}(1-x)u_{1} P(\vartheta _{5}x+(1-\vartheta _{5})\lambda _{5},\vartheta _{i},\lambda _{i})\nonumber \\ & +\frac{1}{2}(1-x)(1-u_{1}) P(\vartheta _{6}x+(1-\vartheta _{6})\lambda _{6},\vartheta _{i},\lambda _{i}) \nonumber \\ & +\frac{1}{2}(1-x)u_{2} P(\vartheta _{7}x+(1-\vartheta _{7})\lambda _{7},\vartheta _{i},\lambda _{i})\nonumber \\ & +\frac{1}{2}(1-x)(1-u_{2}) P(\vartheta _{8}x+(1-\vartheta _{8})\lambda _{8},\vartheta _{i},\lambda _{i}). \end{aligned}$$

## Mathematical analysis

Throughout this work, we let $\mathcal {A}=[0,1]$, $\bar{\mathcal {A} }$ be a nonempty convex subset of a normed space $\mathcal {E}$, $\mathbb {N}$ denotes the set of all positive integers, and the class of all continuous functions $\mathcal {W}:\mathcal {A} \rightarrow \mathbb {R}$ is represented by $\mathcal {B}$ such that $\mathcal {W}(0)=0$ and

$$\begin{aligned} \sup _{\wp _{1} \ne \wp _{2}}\frac{\left| \mathcal {W}(\wp _{1})-\mathcal {W}(\wp _{2})\right| }{\left| \wp _{1} - \wp _{2} \right| }<\infty . \end{aligned}$$

Here, the Banach space associated with $\left( \mathcal {B},\left\| \cdot \right\| \right)$ is evident with

$$\begin{aligned} \left\| \mathcal {W}\right\| =\sup _{\wp _{1} \ne \wp _{2}}\frac{\left| \mathcal {W}(\wp _{1}) - \mathcal {W}(\wp _{2})\right| }{\left| \wp _{1} -\wp _{2} \right| }, \ \ \ \forall \mathcal {W}\in \mathcal {B}. \end{aligned}$$

Furthermore, we rewrite (4.7) as

5.1 $$\begin{aligned} \mathcal {W}(x)= & \frac{1}{2}u_{1}x\mathcal {W}(\vartheta _{1}x+(1-\vartheta _{1})\lambda _{1})\nonumber \\ & +\frac{1}{2}(1-u_{1})x\mathcal {W}(\vartheta _{2}x+(1-\vartheta _{2})\lambda _{2}) \nonumber \\ & +\frac{1}{2}u_{2}x\mathcal {W}(\vartheta _{3}x+(1-\vartheta _{3})\lambda _{3})\nonumber \\ & +\frac{1}{2}(1-u_{2})x\mathcal {W}(\vartheta _{4}x+(1-\vartheta _{4})\lambda _{4}) \nonumber \\ & + \frac{1}{2}(1-x)u_{1} \mathcal {W}(\vartheta _{5}x+(1-\vartheta _{5})\lambda _{5})\nonumber \\ & + \frac{1}{2}(1-x)(1-u_{1}) \mathcal {W}(\vartheta _{6}x+(1-\vartheta _{6})\lambda _{6}) \nonumber \\ & + \frac{1}{2}(1-x)u_{2} \mathcal {W}(\vartheta _{7}x+(1-\vartheta _{7})\lambda _{7})\nonumber \\ & + \frac{1}{2}(1-x)(1-u_{2}) \mathcal {W}(\vartheta _{8}x+(1-\vartheta _{8})\lambda _{8}), \end{aligned}$$

where $0<\vartheta _{1},\vartheta _{2},\vartheta _{3},\vartheta _{4},\vartheta _{5},\vartheta _{6}, \vartheta _{7}, \vartheta _{8}<1$, $\lambda _{k}\ (k=1,2,...,8), u_{1},u_{2}\in \mathcal {A}$, and $\mathcal {W}:\mathcal {A}\rightarrow \mathbb {R}$ is an unknown function.

In the context of analyzing the movement of a rat within a $\mathbb {T}$-maze setup, the parameters described in the functional Eq. (5.1) are interpreted as follows:

1. **State space** $\mathcal {A} = [0,1]$: This parameter defines the range of possible states, where each state $x \in \mathcal {A}$ reflects the probability that the rat will choose a specific direction (left or right) within the maze. A state value near 1 indicates a strong preference for one direction, while a value closer to 0 suggests the opposite.
2. **Learning rate expressions** $\vartheta _{\ell }x + (1 - \vartheta _{\ell }) \lambda _{\ell } \ (\ell = 1, 2, \dots , 8)$: These expressions capture the learning dynamics of the rat. The parameter $\vartheta _{\ell }$ governs how the rat adjusts its behavior based on previous outcomes. A higher value of $\vartheta _{\ell }$ suggests a greater responsiveness to recent experiences, while $\lambda _{\ell }$ represents a baseline fixed point that influences the rat’s preference during the decision-making process.
3. **Decision probability function** $u_{j}x \ (j = 1, 2)$: This function models the probability distribution between the two available choices in the maze: left ($u_1$) or right ($u_2$). It quantifies the likelihood of the rat selecting one of these options. The probabilities evolve over time as part of a Markov decision process, where the state transitions are influenced by the rat’s prior choices and learned behaviors.
4. **Outcome probability function** $\mathcal {W}$: The function $\mathcal {W}$ represents the long-term probability that the rat will consistently choose a particular direction. It reflects how the rat’s decision-making stabilizes over time as it learns from repeated trials, capturing the eventual preference for one path over the other. This function essentially models the culmination of the rat’s learning process, providing insight into its final behavioral patterns after multiple decision-making instances.

Before moving to the main objectives, we define the following key assumptions here, which we shall use in the later section.

($\mathfrak {P}^{\star }$): For an operator $\psi$, there exists a closed subset $\mathcal {O}$ of $\mathcal {B}$ such that $\mathcal {O}$ is $\psi$-invariant, that is, $\psi (\mathcal {O} )\subseteq \mathcal {O}$.

### Theorem 5.1

Let $0<\vartheta _{1},\vartheta _{2},\dots ,\vartheta _{8}<1$ and $\lambda _{k}\ (k=1,2,...,8),u _{1},u _{2} \in \mathcal {A}$ such that $\mathfrak {K}_{1}^{\star } <1,$ where $\mathfrak {K}_{1}^{\star }$ is given in (4.2). If an operator *Z* from $\Lambda$ defined for each $\mathcal {W}$ $\in$ $\Lambda$ and for all $x\in \mathcal {A}$ by

5.2 $$\begin{aligned} (Z\mathcal {W})(x)= & \frac{1}{2}u_{1}x\mathcal {W}(\vartheta _{1}x+(1-\vartheta _{1})\lambda _{1})\nonumber \\ & +\frac{1}{2}(1-u_{1})x\mathcal {W}(\vartheta _{2}x+(1-\vartheta _{2})\lambda _{2}) \nonumber \\ & +\frac{1}{2}u_{2}x\mathcal {W}(\vartheta _{3}x+(1-\vartheta _{3})\lambda _{3})\nonumber \\ & +\frac{1}{2}(1-u_{2})x\mathcal {W}(\vartheta _{4}x+(1-\vartheta _{4})\lambda _{4}) \nonumber \\ & + \frac{1}{2}(1-x)u_{1} \mathcal {W}(\vartheta _{5}x+(1-\vartheta _{5})\lambda _{5})\nonumber \\ & + \frac{1}{2}(1-x)(1-u_{1}) \mathcal {W}(\vartheta _{6}x+(1-\vartheta _{6})\lambda _{6}) \nonumber \\ & + \frac{1}{2}(1-x)u_{2} \mathcal {W}(\vartheta _{7}x+(1-\vartheta _{7})\lambda _{7})\nonumber \\ & + \frac{1}{2}(1-x)(1-u_{2}) \mathcal {W}(\vartheta _{8}x+(1-\vartheta _{8})\lambda _{8}), \end{aligned}$$

and satisfies property ($\mathfrak {P}^{\star }$), then *Z* is a BCM associated with the metric *d* imposed by $\left\| \cdot \right\|$.

### Proof

Let $\mathcal {W}_{1},\mathcal {W}_{2}\in \Lambda$. For all $x,y\in \mathcal {A}$ with $x \ne y$, we have

$$\begin{aligned} & \frac{|(Z\mathcal {W}_{1}-Z\mathcal {W}_{2})(x)-(Z\mathcal {W}_{1}-Z\mathcal {W}_{2})(y)|}{|x-y|} \\= & \bigg |\frac{1}{x-y}\left[ \frac{1}{2}u_{1}x(\mathcal {W}_{1}-\mathcal {W}_{2})(\vartheta _{1}x +(1-\vartheta _{1})\lambda _{1})\right. \\ & +\frac{1}{2}(1-u_{1})x(\mathcal {W}_{1}-\mathcal {W}_{2})(\vartheta _{2}x+(1-\vartheta _{2})\lambda _{2}) \\ & +\frac{1}{2}u_{2}x(\mathcal {W}_{1}-\mathcal {W}_{2})(\vartheta _{3}x+(1-\vartheta _{3})\lambda _{3})\\ & +\frac{1}{2}(1-u_{2})x(\mathcal {W}_{1}-\mathcal {W}_{2})(\vartheta _{4}x+(1-\vartheta _{4})\lambda _{4}) \\ & +\frac{1}{2}(1-x)u_{1} (\mathcal {W}_{1}-\mathcal {W}_{2})(\vartheta _{5}x+(1-\vartheta _{5})\lambda _{5})\\ & + \frac{1}{2}(1-x)(1-u_{1}) (\mathcal {W}_{1}-\mathcal {W}_{2})(\vartheta _{6}x+(1-\vartheta _{6})\lambda _{6}) \\ & + \frac{1}{2}(1-x)u_{2} (\mathcal {W}_{1}-\mathcal {W}_{2})(\vartheta _{7}x+(1-\vartheta _{7})\lambda _{7})\\ & + \frac{1}{2}(1-x)(1-u_{2}) (\mathcal {W}_{1}-\mathcal {W}_{2})(\vartheta _{8}x+(1-\vartheta _{8})\lambda _{8}) \\ & -\frac{1}{2}u_{1}y(\mathcal {W}_{1}-\mathcal {W}_{2})(\vartheta _{1}y+(1-\vartheta _{1})\lambda _{1})\\ & -\frac{1}{2}(1-u_{1})y(\mathcal {W}_{1}-\mathcal {W}_{2})(\vartheta _{2}y+(1-\vartheta _{2})\lambda _{2}) \\ & -\frac{1}{2}u_{2}y(\mathcal {W}_{1}-\mathcal {W}_{2})(\vartheta _{3}y+(1-\vartheta _{3})\lambda _{3})\\ & -\frac{1}{2}(1-u_{2})y(\mathcal {W}_{1}-\mathcal {W}_{2})(\vartheta _{4}y+(1-\vartheta _{4})\lambda _{4}) \\ & -\frac{1}{2}(1-y)u_{1} (\mathcal {W}_{1}-\mathcal {W}_{2})(\vartheta _{5}y+(1-\vartheta _{5})\lambda _{5})\\ & - \frac{1}{2}(1-y)(1-u_{1}) (\mathcal {W}_{1}-\mathcal {W}_{2})(\vartheta _{6}y+(1-\vartheta _{6})\lambda _{6}) \\ & - \frac{1}{2}(1-y)u_{2} (\mathcal {W}_{1}-\mathcal {W}_{2})(\vartheta _{7}y+(1-\vartheta _{7})\lambda _{7})\\ & \left. - \frac{1}{2}(1-y)(1-u_{2}) (\mathcal {W}_{1}-\mathcal {W}_{2})(\vartheta _{8}y+(1-\vartheta _{8})\lambda _{8})\right] \bigg | \\= & \bigg |\frac{1}{x-y}\left[ \frac{1}{2}u_{1}x(\mathcal {W}_{1}-\mathcal {W}_{2})(\vartheta _{1}x+(1-\vartheta _{1})\lambda _{1})\right. \\ & -\frac{1}{2}u_{1}x(\mathcal {W}_{1}-\mathcal {W}_{2})(\vartheta _{1}y+(1-\vartheta _{1})\lambda _{1})\\ & +\frac{1}{2}(1-u_{1})x(\mathcal {W}_{1}-\mathcal {W}_{2})(\vartheta _{2}x+(1-\vartheta _{2})\lambda _{2})\\ & -\frac{1}{2}(1-u_{1})x(\mathcal {W}_{1}-\mathcal {W}_{2})(\vartheta _{2}y+(1-\vartheta _{2})\lambda _{2}) \\ & +\frac{1}{2}u_{2}x(\mathcal {W}_{1}-\mathcal {W}_{2})(\vartheta _{3}x+(1-\vartheta _{3})\lambda _{3})\\ & -\frac{1}{2}u_{2}x(\mathcal {W}_{1}-\mathcal {W}_{2})(\vartheta _{3}y+(1-\vartheta _{3})\lambda _{3}) \\ & +\frac{1}{2}(1-u_{2})x(\mathcal {W}_{1}-\mathcal {W}_{2})(\vartheta _{4}x+(1-\vartheta _{4})\lambda _{4})\\ & -\frac{1}{2}(1-u_{2})x(\mathcal {W}_{1}-\mathcal {W}_{2})(\vartheta _{4}y+(1-\vartheta _{4})\lambda _{4}) \\ & + \frac{1}{2}(1-x)u_{1} (\mathcal {W}_{1}-\mathcal {W}_{2})(\vartheta _{5}x+(1-\vartheta _{5})\lambda _{5})\\ & -\frac{1}{2}(1-x)u_{1} (\mathcal {W}_{1}-\mathcal {W}_{2})(\vartheta _{5}y+(1-\vartheta _{5})\lambda _{5}) \end{aligned}$$

$$\begin{aligned} & + \frac{1}{2}(1-y)(1-u_{1}) (\mathcal {W}_{1}-\mathcal {W}_{2})(\vartheta _{6}x+(1-\vartheta _{6})\lambda _{6})\\ & - \frac{1}{2}(1-x)(1-u_{1}) (\mathcal {W}_{1}-\mathcal {W}_{2})(\vartheta _{6}y+(1-\vartheta _{6})\lambda _{6}) \\ & + \frac{1}{2}(1-x)u_{2} (\mathcal {W}_{1}-\mathcal {W}_{2})(\vartheta _{7}x+(1-\vartheta _{7})\lambda _{7})\\ & - \frac{1}{2}(1-x)u_{2} (\mathcal {W}_{1}-\mathcal {W}_{2})(\vartheta _{7}y+(1-\vartheta _{7})\lambda _{7}) \\ & + \frac{1}{2}(1-x)(1-u_{2}) (\mathcal {W}_{1}-\mathcal {W}_{2})(\vartheta _{8}x+(1-\vartheta _{8})\lambda _{8})\\ & - \frac{1}{2}(1-x)(1-u_{2}) (\mathcal {W}_{1}-\mathcal {W}_{2})(\vartheta _{8}y+(1-\vartheta _{8})\lambda _{8}) \\ & +\frac{1}{2}u_{1}x(\mathcal {W}_{1}-\mathcal {W}_{2})(\vartheta _{1}y+(1-\vartheta _{1})\lambda _{1})\\ & -\frac{1}{2}u_{1}y(\mathcal {W}_{1}- \mathcal {W}_{2})(\vartheta _{1}y+(1-\vartheta _{1})\lambda _{1}) \\ & +\frac{1}{2}(1-u_{1})x(\mathcal {W}_{1}-\mathcal {W}_{2})(\vartheta _{2}y+(1-\vartheta _{2})\lambda _{2})\\ & -\frac{1}{2}(1-u_{1})y(\mathcal {W}_{1}-\mathcal {W}_{2})(\vartheta _{2}y+(1-\vartheta _{2})\lambda _{2}) \\ & +\frac{1}{2}u_{2}x(\mathcal {W}_{1}-\mathcal {W}_{2})(\vartheta _{3}y+(1-\vartheta _{3})\lambda _{3})\\ & -\frac{1}{2}u_{2}y(\mathcal {W}_{1}-\mathcal {W}_{2})(\vartheta _{3}y+(1-\vartheta _{3})\lambda _{3}) \\ & +\frac{1}{2}(1-u_{2})x(\mathcal {W}_{1}-\mathcal {W}_{2})(\vartheta _{4}y+(1-\vartheta _{4})\lambda _{4})\\ & -\frac{1}{2}(1-u_{2})y(\mathcal {W}_{1}-\mathcal {W}_{2})(\vartheta _{4}y+(1-\vartheta _{4})\lambda _{4}) \\ & +\frac{1}{2}(1-x)u_{1} (\mathcal {W}_{1}-\mathcal {W}_{2})(\vartheta _{5}y+(1-\vartheta _{5})\lambda _{5})\\ & -\frac{1}{2}(1-x)u_{1} (\mathcal {W}_{1}-\mathcal {W}_{2})(\vartheta _{5}y+(1-\vartheta _{5})\lambda _{5}) \\ & + \frac{1}{2}(1-x)(1-u_{1}) (\mathcal {W}_{1}-\mathcal {W}_{2})(\vartheta _{6}y+(1-\vartheta _{6})\lambda _{6})\\ & -\frac{1}{2}(1-x)(1-u_{1}) (\mathcal {W}_{1}-\mathcal {W}_{2})(\vartheta _{6}y+(1-\vartheta _{6})\lambda _{6}) \\ & + \frac{1}{2}(1-x)u_{2} (\mathcal {W}_{1}-\mathcal {W}_{2})(\vartheta _{7}y+(1-\vartheta _{7})\lambda _{7})\\ & - \frac{1}{2}(1-y)u_{2} (\mathcal {W}_{1}-\mathcal {W}_{2})(\vartheta _{7}y+(1-\vartheta _{7})\lambda _{7}) \\ & + \frac{1}{2}(1-x)(1-u_{2}) (\mathcal {W}_{1}-\mathcal {W}_{2})(\vartheta _{8}y+(1-\vartheta _{8})\lambda _{8})\\ & \left. - \frac{1}{2}(1-y)(1-u_{2}) (\mathcal {W}_{1}-\mathcal {W}_{2})(\vartheta _{8}y+(1-\vartheta _{8})\lambda _{8}) \right] \bigg | \end{aligned}$$

$$\begin{aligned}= & \bigg | \frac{1}{x-y}\left[ \frac{1}{2}u_{1}x(\mathcal {W}_{1}-\mathcal {W}_{2})(\vartheta _{1}x+(1-\vartheta _{1})\lambda _{1})\right. \\ & \left. -\frac{1}{2}u_{1}x(\mathcal {W}_{1}-\mathcal {W}_{2})(\vartheta _{1}y+(1-\vartheta _{1})\lambda _{1}) \right] \\ & +\frac{1}{x-y}\left[ \frac{1}{2}(1-u_{1})x(\mathcal {W}_{1}-\mathcal {W}_{2})(\vartheta _{2}x+(1-\vartheta _{2})\lambda _{2})\right. \\ & \left. -\frac{1}{2}(1-u_{1})x(\mathcal {W}_{1}-\mathcal {W}_{2})(\vartheta _{2}y+(1-\vartheta _{2})\lambda _{2}) \right] \\ & +\frac{1}{x-y}\left[ \frac{1}{2}u_{2}x(\mathcal {W}_{1}-\mathcal {W}_{2})(\vartheta _{3}x+(1-\vartheta _{3})\lambda _{3})\right. \\ & \left. -\frac{1}{2}u_{2}x(\mathcal {W}_{1}-\mathcal {W}_{2})(\vartheta _{3}y+(1-\vartheta _{3})\lambda _{3}) \right] \\ & +\frac{1}{x-y}\left[ \frac{1}{2}(1-u_{2})x(\mathcal {W}_{1}-\mathcal {W}_{2})(\vartheta _{4}x+(1-\vartheta _{4})\lambda _{4})\right. \\ & \left. -\frac{1}{2}(1-u_{2})x(\mathcal {W}_{1}-\mathcal {W}_{2})(\vartheta _{4}y+(1-\vartheta _{4})\lambda _{4}) \right] \\ & +\frac{1}{x-y}\left[ \frac{1}{2}(1-x)u_{1} (\mathcal {W}_{1}-\mathcal {W}_{2})(\vartheta _{5}x+(1-\vartheta _{5})\lambda _{5})\right. \\ & \left. -\frac{1}{2}(1-x)u_{1} (\mathcal {W}_{1}-\mathcal {W}_{2})(\vartheta _{5}y+(1-\vartheta _{5})\lambda _{5}) \right] \\ & +\frac{1}{x-y}\left[ \frac{1}{2}(1-y)(1-u_{1}) (\mathcal {W}_{1}-\mathcal {W}_{2})(\vartheta _{6}x+(1-\vartheta _{6})\lambda _{6})\right. \\ & \left. - \frac{1}{2}(1-x)(1-u_{1}) (\mathcal {W}_{1}-\mathcal {W}_{2})(\vartheta _{6}y+(1-\vartheta _{6})\lambda _{6}) \right] \\ & +\frac{1}{x-y}\left[ \frac{1}{2}(1-x)u_{2} (\mathcal {W}_{1}-\mathcal {W}_{2})(\vartheta _{7}x+(1-\vartheta _{7})\lambda _{7})\right. \\ & \left. - \frac{1}{2}(1-x)u_{2} (\mathcal {W}_{1}-\mathcal {W}_{2})(\vartheta _{7}y+(1-\vartheta _{7})\lambda _{7}) \right] \\ & +\frac{1}{x-y}\left[ \frac{1}{2}(1-x)(1-u_{2}) (\mathcal {W}_{1}-\mathcal {W}_{2})(\vartheta _{8}x+(1-\vartheta _{8})\lambda _{8})\right. \\ & \left. - \frac{1}{2}(1-x)(1-u_{2}) (\mathcal {W}_{1}-\mathcal {W}_{2})(\vartheta _{8}y+(1-\vartheta _{8})\lambda _{8})\right] \\ & +\frac{1}{x-y}\left[ \frac{1}{2}u_{1}x(\mathcal {W}_{1}-\mathcal {W}_{2})(\vartheta _{1}y+(1-\vartheta _{1})\lambda _{1})\right. \\ & \left. -\frac{1}{2}u_{1}y(\mathcal {W}_{1}- \mathcal {W}_{2})(\vartheta _{1}y+(1-\vartheta _{1})\lambda _{1}) \right] \\ & +\frac{1}{x-y}\left[ \frac{1}{2}(1-u_{1})x(\mathcal {W}_{1}-\mathcal {W}_{2})(\vartheta _{2}y+(1-\vartheta _{2})\lambda _{2})\right. \\ & \left. -\frac{1}{2}(1-u_{1})y(\mathcal {W}_{1}-\mathcal {W}_{2})(\vartheta _{2}y+(1-\vartheta _{2})\lambda _{2}) \right] \\ & +\frac{1}{x-y}\left[ \frac{1}{2}u_{2}x(\mathcal {W}_{1}-\mathcal {W}_{2})(\vartheta _{3}y+(1-\vartheta _{3})\lambda _{3})\right. \\ & \left. -\frac{1}{2}u_{2}y(\mathcal {W}_{1}-\mathcal {W}_{2})(\vartheta _{3}y+(1-\vartheta _{3})\lambda _{3}) \right] \end{aligned}$$

$$\begin{aligned} & +\frac{1}{x-y}\left[ \frac{1}{2}(1-u_{2})x(\mathcal {W}_{1}-\mathcal {W}_{2})(\vartheta _{4}y+(1-\vartheta _{4})\lambda _{4})\right. \\ & \left. -\frac{1}{2}(1-u_{2})y(\mathcal {W}_{1}-\mathcal {W}_{2})(\vartheta _{4}y+(1-\vartheta _{4})\lambda _{4}) \right] \\ & +\frac{1}{x-y}\left[ \frac{1}{2}(1-x)u_{1} (\mathcal {W}_{1}-\mathcal {W}_{2})(\vartheta _{5}y+(1-\vartheta _{5})\lambda _{5})\right. \\ & \left. -\frac{1}{2}(1-y)u_{1} (\mathcal {W}_{1}-\mathcal {W}_{2})(\vartheta _{5}y+(1-\vartheta _{5})\lambda _{5}) \right] \\ & +\frac{1}{x-y}\left[ \frac{1}{2}(1-x)(1-u_{1}) (\mathcal {W}_{1}-\mathcal {W}_{2})(\vartheta _{6}y+(1-\vartheta _{6})\lambda _{6})\right. \\ & \left. -\frac{1}{2}(1-y)(1-u_{1}) (\mathcal {W}_{1}-\mathcal {W}_{2})(\vartheta _{6}y+(1-\vartheta _{6})\lambda _{6}) \right] \\ & +\frac{1}{x-y}\left[ \frac{1}{2}(1-x)u_{2} (\mathcal {W}_{1}-\mathcal {W}_{2})(\vartheta _{7}y+(1-\vartheta _{7})\lambda _{7})\right. \\ & \left. - \frac{1}{2}(1-y)u_{2} (\mathcal {W}_{1}-\mathcal {W}_{2})(\vartheta _{7}y+(1-\vartheta _{7})\lambda _{7}) \right] \\ & +\frac{1}{x-y}\left[ \frac{1}{2}(1-x)(1-u_{2}) (\mathcal {W}_{1}-\mathcal {W}_{2})(\vartheta _{8}y+(1-\vartheta _{8})\lambda _{8})\right. \\ & \left. - \frac{1}{2}(1-y)(1-u_{2}) (\mathcal {W}_{1}-\mathcal {W}_{2})(\vartheta _{8}y+(1-\vartheta _{8})\lambda _{8}) \right] \bigg | \\\le & \frac{1}{2}u_{1}x\vartheta _{1}\Vert \mathcal {W}_{1}-\mathcal {W}_{2}\Vert \\ & +\frac{1}{2}(1-u_{1})x\vartheta _{2}\Vert \mathcal {W}_{1}-\mathcal {W}_{2}\Vert +\frac{1}{2}u_{2}x\vartheta _{3}\Vert \mathcal {W}_{1}-\mathcal {W}_{2}\Vert \\ & +\frac{1}{2}(1-u_{2})x\vartheta _{4}\Vert \mathcal {W}_{1}-\mathcal {W}_{2}\Vert \\ & + \frac{1}{2}(1-x) u_{1}\vartheta _{5}\Vert \mathcal {W}_{1}-\mathcal {W}_{2}\Vert \\ & + \frac{1}{2}(1-x)(1-u_{1}) \vartheta _{6}\Vert \mathcal {W}_{1}-\mathcal {W}_{2}\Vert \\ & + \frac{1}{2}(1-x) u_{2}\vartheta _{7}\Vert \mathcal {W}_{1}-\mathcal {W}_{2}\Vert \\ & + \frac{1}{2}(1-x)(1-u_{2})\vartheta _{8}\Vert \mathcal {W}_{1}-\mathcal {W}_{2}\Vert \\ & +\left| \frac{1}{2}u_{1}(\mathcal {W}_{1}-\mathcal {W}_{2})(\vartheta _{1}y+(1-\vartheta _{1})\lambda _{1})\right. \\ & \left. -\frac{1}{2}u_{1}(\mathcal {W}_{1}-\mathcal {W}_{2})(0)\right| \\ & +\left| \frac{1}{2}(1-u_{1})(\mathcal {W}_{1}-\mathcal {W}_{2})(\vartheta _{2}y+(1-\vartheta _{2})\lambda _{2}) -\frac{1}{2}(1-u_{1})(\mathcal {W}_{1}-\mathcal {W}_{2})(0)\right| \\ & +\left| \frac{1}{2}u_{2}(\mathcal {W}_{1}-\mathcal {W}_{2})(\vartheta _{3}y+(1-\vartheta _{3})\lambda _{3})\right. \\ & \left. -\frac{1}{2}u_{2}(\mathcal {W}_{1}-\mathcal {W}_{2})(0)\right| \\ & +\left| \frac{1}{2}(1-u_{2})(\mathcal {W}_{1}-\mathcal {W}_{2})(\vartheta _{4}y+(1-\vartheta _{4})\lambda _{4})\right. \\ & \left. -\frac{1}{2}(1-u_{2})(\mathcal {W}_{1}-\mathcal {W}_{2})(0)\right| \\ & +\left| \frac{1}{2}(\mathcal {W}_{1}-\mathcal {W}_{2})(\vartheta _{5}y+(1-\vartheta _{5})\lambda _{5})\right. \\ & \left. -\frac{1}{2}(\mathcal {W}_{1}-\mathcal {W}_{2})(0)\right| \end{aligned}$$

$$\begin{aligned} & +\left| \frac{1}{2}(\mathcal {W}_{1}-\mathcal {W}_{2})(\vartheta _{6}y+(1-\vartheta _{6})\lambda _{6}) -\frac{1}{2}(\mathcal {W}_{1}-\mathcal {W}_{2})(0)\right| \\ & +\left| \frac{1}{2}(\mathcal {W}_{1}-\mathcal {W}_{2})(\vartheta _{7}y+(1-\vartheta _{7})\lambda _{7}) -\frac{1}{2}(\mathcal {W}_{1}-\mathcal {W}_{2})(0)\right| \\ & +\left| \frac{1}{2}(\mathcal {W}_{1}-\mathcal {W}_{2})(\vartheta _{8}y+(1-\vartheta _{8})\lambda _{8}) -\frac{1}{2}(\mathcal {W}_{1}-\mathcal {W}_{2})(0)\right| \\\le & \frac{1}{2}u_{1} \vartheta _{1}\Vert \mathcal {W}_{1}-\mathcal {W}_{2}\Vert +\frac{1}{2}(1-u_{1})\vartheta _{2}\Vert \mathcal {W}_{1}-\mathcal {W}_{2}\Vert \\ & +\frac{1}{2}u_{2}\vartheta _{3}\Vert \mathcal {W}_{1}-\mathcal {W}_{2}\Vert +\frac{1}{2}(1-u_{2})\vartheta _{4}\Vert \mathcal {W}_{1}-\mathcal {W}_{2}\Vert \\ & \frac{1}{2}u_{1} \vartheta _{5}\Vert \mathcal {W}_{1}-\mathcal {W}_{2}\Vert +\frac{1}{2}(1-u_{1})\vartheta _{6}\Vert \mathcal {W}_{1}-\mathcal {W}_{2}\Vert \\ & +\frac{1}{2}u_{2}\vartheta _{7}\Vert \mathcal {W}_{1}-\mathcal {W}_{2}\Vert +\frac{1}{2}(1-u_{2})\vartheta _{8}\Vert \mathcal {W}_{1}-\mathcal {W}_{2}\Vert \\ & +\frac{1}{2}u_{1}(\vartheta _{1}+(1-\vartheta _{1})\lambda _{1})\Vert \mathcal {W} _{1}-\mathcal {W}_{2}\Vert \\ & +\frac{1}{2} (1- u_{1}) (\vartheta _{2}+(1-\vartheta _{2})\lambda _{2})\Vert \mathcal {W}_{1}-\mathcal {W}_{2}\Vert \\ & +\frac{1}{2}u_{2}(\vartheta _{3}+(1-\vartheta _{3})\lambda _{3})\Vert \mathcal {W} _{1}-\mathcal {W}_{2}\Vert \\ & +\frac{1}{2} (1- u_{2}) (\vartheta _{4}+(1-\vartheta _{4})\lambda _{4})\Vert \mathcal {W}_{1}-\mathcal {W}_{2}\Vert \\ & +\frac{1}{2}u_{1}(\vartheta _{5}+(1-\vartheta _{5})\lambda _{5})\Vert \mathcal {W} _{5}-\mathcal {W}_{2}\Vert \\ & +\frac{1}{2} (1- u_{1}) (\vartheta _{6}+(1-\vartheta _{6})\lambda _{6})\Vert \mathcal {W}_{1}-\mathcal {W}_{2}\Vert \\ & +\frac{1}{2}u_{2}(\vartheta _{7}+(1-\vartheta _{7})\lambda _{7})\Vert \mathcal {W}_{1}-\mathcal {W}_{2}\Vert \\ & +\frac{1}{2} (1- u_{2}) (\vartheta _{8}+(1-\vartheta _{8})\lambda _{8})\Vert \mathcal {W}_{1}-\mathcal {W}_{2}\Vert \quad \le \mathfrak {K}_{1}^{\star }\Vert \mathcal {W}_{1}-\mathcal {W}_{2}\Vert . \end{aligned}$$

This gives that

$$\begin{aligned} d(Z\mathcal {W}_{1},Z\mathcal {W}_{2})= & \Vert Z\mathcal {W}_{1}-Z\mathcal {W}_{2}\Vert \\\le & \mathfrak {K}_{1}^{\star } \Vert \mathcal {W}_{1}-\mathcal {W}_{2}\Vert \\= & \mathfrak {K}_{1}^{\star } d(\mathcal {W}_{1},\mathcal {W}_{2}). \end{aligned}$$

As $0<\mathfrak {K}_{1}^{\star } <1,$ hence *Z* is a BCM with the metric *d* imposed by $\left\| \cdot \right\|$. $\square$

From Theorem 5.1, we may deduce the following regarding the unique solution of a stochastic Eq. (5.1).

### Theorem 5.2

The functional Eq. (5.1) has a unique solution provided that $\mathfrak {K}_{1}^{\star }<1,$ where $\mathfrak {K}_{1}^{\star }$ is defined by (4.2), and there exists an operator *Z* from $\Lambda$ defined for each $\mathcal {W}$ $\in \Lambda$ and for all $x\in \mathcal {A}$ in (5.2) and satisfies property ($\mathfrak {P}^{\star }$). Furthermore, the iteration $\{\mathcal {W}_{n}\}$ ($\forall n\in \mathbb {N}$) in $\Lambda$, where $\mathcal {W}_{0}\in \Lambda$, is given by

5.3 $$\begin{aligned} (\mathcal {W}_{n})(x)= & \frac{1}{2}u_{1}x\mathcal {W}_{n-1}(\vartheta _{1}x+(1-\vartheta _{1})\lambda _{1})\nonumber \\ & +\frac{1}{2}(1-u_{1})x\mathcal {W}_{n-1}(\vartheta _{2}x+(1-\vartheta _{2})\lambda _{2}) \nonumber \\ & +\frac{1}{2}u_{2}x\mathcal {W}_{n-1}(\vartheta _{3}x+(1-\vartheta _{3})\lambda _{3})\nonumber \\ & +\frac{1}{2}(1-u_{2})x\mathcal {W}_{n-1}(\vartheta _{4}x+(1-\vartheta _{4})\lambda _{4}) \nonumber \\ & + \frac{1}{2}(1-x)u_{1} \mathcal {W}_{n-1}(\vartheta _{5}x+(1-\vartheta _{5})\lambda _{5})\nonumber \\ & + \frac{1}{2}(1-x)(1 - u_{1}) \mathcal {W}_{n-1}(\vartheta _{6}x+(1-\vartheta _{6})\lambda _{6}), \nonumber \\ & + \frac{1}{2}(1-x)u_{2} \mathcal {W}_{n-1}(\vartheta _{7}x+(1-\vartheta _{7})\lambda _{7})\nonumber \\ & + \frac{1}{2}(1-x)(1 - u_{2}) \mathcal {W}_{n-1}(\vartheta _{8}x+(1-\vartheta _{8})\lambda _{8}), \end{aligned}$$

converges to the unique solution of the stochastic Eq. (5.1).

### Proof

We conclude the proof by coupling Theorem 5.1 with the Banach fixed point theorem. $\square$

### Asymptotic behavior and numerical approximation

**Fig. 7:**
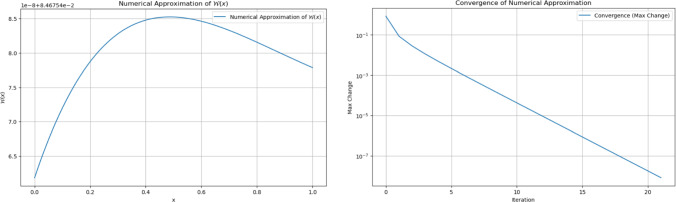
Numerical approximation and convergence of $\mathcal {W}(x)$

The numerical behavior of $\mathcal {W}(x)$ is analyzed through a combination of iterative approximations and asymptotic evaluations. Figure 7, observed with specific parameter settings ($\theta$ and $\lambda$ values), illustrates the function’s convergence properties and numerical stability. The iterative method demonstrates a consistent reduction in the maximum change between successive approximations, affirming the reliability of the numerical scheme employed. Additionally, the function exhibits a characteristic trend across its domain, initially rising before reaching a peak and subsequently declining, reinforcing its nonlinear structure under the defined parameter constraints.

**Fig. 8:**
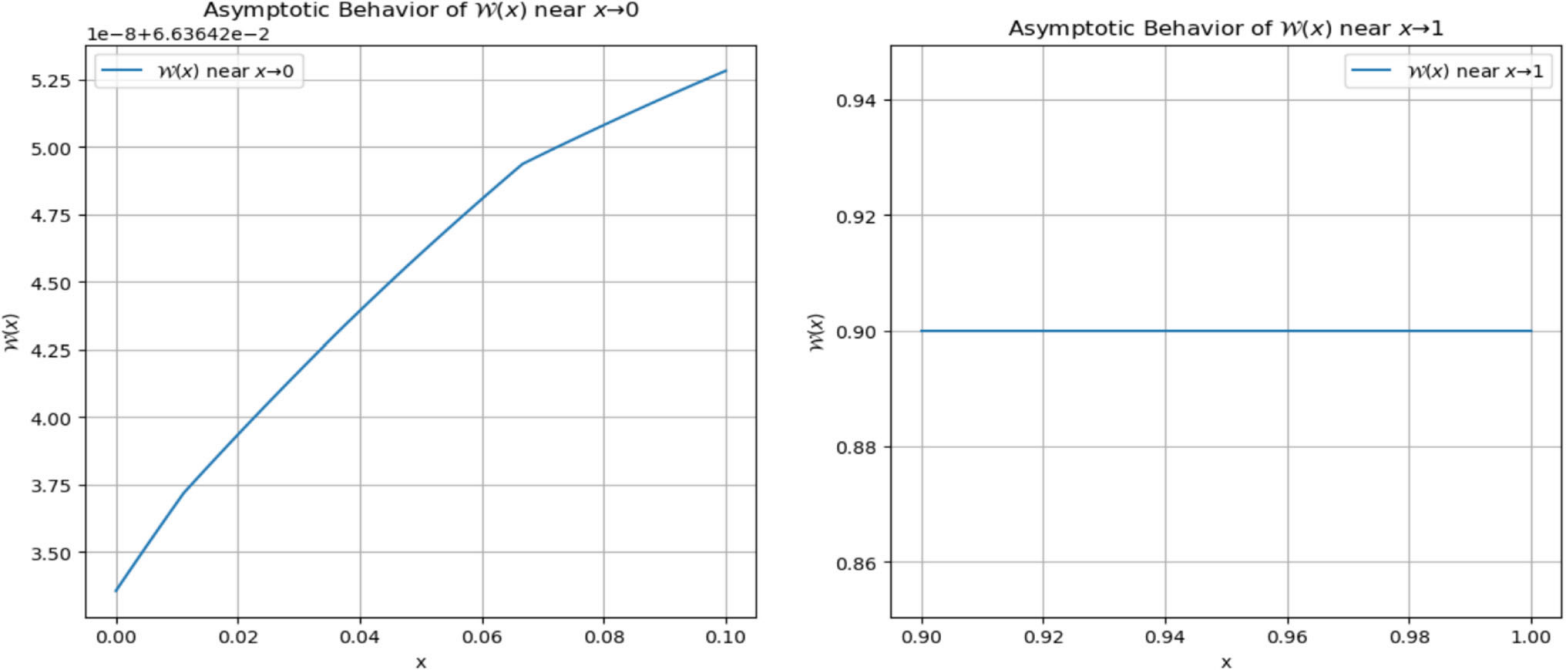
Asymptotic behavior of $\mathcal {W}(x)$ as *x* approaches 0 and 1

The asymptotic properties of $\mathcal {W}(x)$ as $x$ approaches the boundaries provide further insights into its stability. Figure 8 reveals that near $x=0$, the function experiences steady growth, stabilizing without divergence, while at $x=1$, it remains relatively constant, suggesting minimal variation. These observations align with theoretical expectations, supporting the existence and uniqueness of solutions derived via the Banach fixed-point theorem. Such graphical insights validate the robustness of the functional equation and its sensitivity to initial conditions.

**Fig. 9:**
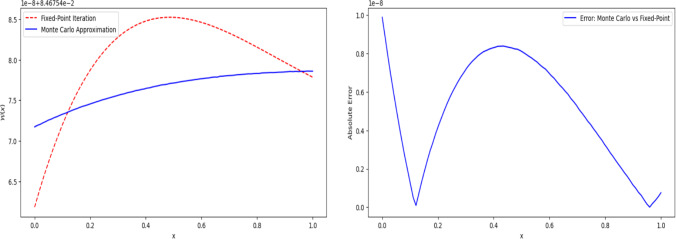
Comparison and error analysis of fixed-point and Monte Carlo approximations

To further evaluate the numerical approach, we compare the fixed-point iteration method with the Monte Carlo approximation and their error analysis (see Fig. 9). The fixed-point method exhibits smooth deterministic convergence, ensuring well-defined solutions, while the Monte Carlo approach, leveraging stochastic sampling, introduces slight deviations. The latter closely follows the deterministic trajectory but tends to underestimate peak values due to its inherent randomness. Error analysis indicates that while absolute error remains within an acceptable range, fluctuations occur around mid-domain values before gradually diminishing. This trade-off highlights the precision of fixed-point methods in solving functional equations while acknowledging the probabilistic variability introduced by Monte Carlo approximations.

## Some specific features

In this portion, certain situations of the Wyckoff stochastic model have been examined.

### Situation with identical lambda constraints

This condition, also known as the commutative condition, states that our transition operators $P_{1}$ through $P_{6}$ (none of which are identity operators) have identical lambda conditions $(\lambda _{1}=\lambda _{2}=\lambda _{3}=\lambda _{4}=\lambda _{5}=\lambda _{6}=\lambda _{7}=\lambda _{8}=\lambda )$. Due to these constraints, our transition operators (4.6) are reduced to

6.1 $$\begin{aligned} \left\{ \begin{aligned} P_{1}x&= \vartheta _{1}x+(1-\vartheta _{1})\lambda , \\ P_{2}x&=\vartheta _{2}x+(1-\vartheta _{2})\lambda , \\ P_{3}x&=\vartheta _{3}x+(1-\vartheta _{3})\lambda , \\ P_{4}x&=\vartheta _{4}x+(1-\vartheta _{4})\lambda , \\ P_{5}x&=\vartheta _{5}x+(1-\vartheta _{5})\lambda , \\ P_{6}x&=\vartheta _{6}x+(1-\vartheta _{6})\lambda , \\ P_{7}x&=\vartheta _{7}x+(1-\vartheta _{7})\lambda , \\ P_{8}x&=\vartheta _{8}x+(1-\vartheta _{8})\lambda . \ \end{aligned}\right. \end{aligned}$$

By ensuring that all transition operators share the same lambda value, this condition maintains the model’s stability across different maze structures and configurations. This uniformity allows the model to generalize beyond a specific experimental setting, making it adaptable to alternative maze layouts without requiring extensive parameter adjustments. Now, our functional Eq. (5.1) can be expressed as

6.2 $$\begin{aligned} \mathcal {W}(x)= & \frac{1}{2}u_{1}x\mathcal {W}(\vartheta _{1}x+(1-\vartheta _{1})\lambda )\nonumber \\ & +\frac{1}{2}(1-u_{1})x\mathcal {W}(\vartheta _{2}x+(1-\vartheta _{2})\lambda ) \nonumber \\ & +\frac{1}{2}u_{2}x\mathcal {W}(\vartheta _{3}x+(1-\vartheta _{3})\lambda )\nonumber \\ & +\frac{1}{2}(1-u_{2})x\mathcal {W}(\vartheta _{4}x+(1-\vartheta _{4})\lambda ) \nonumber \\ & +\frac{1}{2}(1-x)u_{1} \mathcal {W}(\vartheta _{5}x+(1-\vartheta _{5})\lambda )\nonumber \\ & + \frac{1}{2}(1-x)(1 - u_{1}) \mathcal {W}(\vartheta _{6}x+(1-\vartheta _{6})\lambda ), \nonumber \\ & +\frac{1}{2}(1-x)u_{2} \mathcal {W}(\vartheta _{7}x+(1-\vartheta _{7})\lambda )\nonumber \\ & + \frac{1}{2}(1-x)(1 - u_{2}) \mathcal {W}(\vartheta _{8}x+(1-\vartheta _{8})\lambda ), \end{aligned}$$

where $0<\vartheta _{1},\vartheta _{2},\vartheta _{3},\vartheta _{4},\vartheta _{5},\vartheta _{6}, \vartheta _{7}, \vartheta _{8}<1$, $\lambda ,u_{1},u_{2}\in \mathcal {A}$ and $\mathcal {W}:\mathcal {A}\rightarrow \mathbb {R}$ is an unknown function. The Theorem 5.1 has the following findings as a result.

#### Corollary 6.1

Let $0<\vartheta _{1},\vartheta _{2},\dots ,\vartheta _{8}<1$ and $\lambda ,u_{1},u_{2}\in \mathcal {A}$ with $\mathfrak {K}_{2}^{\star } <1$, where $\mathfrak {K}_{2}^{\star }$ is defined in (4.3). If an operator *Z* from $\Lambda$ associated for every $\mathcal {W}$ $\in$ $\Lambda$ and for all $x\in \mathcal {A}$ by

6.3 $$\begin{aligned} (Z\mathcal {W})(x)= & \frac{1}{2}u_{1}x\mathcal {W}(\vartheta _{1}x+(1-\vartheta _{1})\lambda )\nonumber \\ & +\frac{1}{2}(1-u_{1})x\mathcal {W}(\vartheta _{2}x+(1-\vartheta _{2})\lambda ) \nonumber \\ & +\frac{1}{2}u_{2}x\mathcal {W}(\vartheta _{3}x+(1-\vartheta _{3})\lambda ) \nonumber \\ & + \frac{1}{2}(1-u_{2})x\mathcal {W}(\vartheta _{4}x+(1-\vartheta _{4})\lambda ) \nonumber \\ & +\frac{1}{2}(1-x)u_{1} \mathcal {W}(\vartheta _{5}x+(1-\vartheta _{5})\lambda )\nonumber \\ & + \frac{1}{2}(1-x)(1 - u_{1}) \mathcal {W}(\vartheta _{6}x+(1-\vartheta _{6})\lambda ), \nonumber \\ & +\frac{1}{2}(1-x)u_{2} \mathcal {W}(\vartheta _{7}x+(1-\vartheta _{7})\lambda )\nonumber \\ & + \frac{1}{2}(1-x)(1 - u_{2}) \mathcal {W}(\vartheta _{8}x+(1-\vartheta _{8})\lambda ), \end{aligned}$$

and satisfies property ($\mathfrak {P}^{\star }$), then *Z* is a BCM.

#### Corollary 6.2

The Eq. (6.2) has a unique solution having that $\mathfrak {K}_{2}^{\star } <1$, where $\mathfrak {K}_{2}^{\star }$ is given in (4.3), and there exists an operator *Z* defined in (6.3) satisfies property ($\mathfrak {P}^{\star }$). Furthermore, the iteration $\{\mathcal {W}_{n}\}$ ($\forall n\in \mathbb {N}$) in $\Lambda$, where $\mathcal {W}_{0}\in \Lambda$, is given by

6.4 $$\begin{aligned} (\mathcal {W}_{n})(x)= & \frac{1}{2}u_{1}x\mathcal {W}_{n-1}(\vartheta _{1}x+(1-\vartheta _{1})\lambda )\nonumber \\ & +\frac{1}{2}(1-u_{1})x\mathcal {W}_{n-1}(\vartheta _{2}x+(1-\vartheta _{2})\lambda ) \nonumber \\ & +\frac{1}{2}u_{2}x\mathcal {W}_{n-1}(\vartheta _{3}x+(1-\vartheta _{3})\lambda )\nonumber \\ & +\frac{1}{2}(1-u_{2})x\mathcal {W}_{n-1}(\vartheta _{4}x+(1-\vartheta _{4})\lambda ) \nonumber \\ & +\frac{1}{2}(1-x)u_{1} \mathcal {W}_{n-1}(\vartheta _{5}x+(1-\vartheta _{5})\lambda )\nonumber \\ & + \frac{1}{2}(1-x)(1 - u_{1}) \mathcal {W}_{n-1}(\vartheta _{6}x+(1-\vartheta _{6})\lambda ),\nonumber \\ & +\frac{1}{2}(1-x)u_{2} \mathcal {W}_{n-1}(\vartheta _{7}x+(1-\vartheta _{7})\lambda )\nonumber \\ & + \frac{1}{2}(1-x)(1 - u_{2}) \mathcal {W}_{n-1}(\vartheta _{8}x+(1-\vartheta _{8})\lambda ), \end{aligned}$$

converges to the unique solution of (6.2).

### Elimination of a behavioral reflex

It’s possible that the mouse’s frequent right- or left-turning side reactions might reduce an event’s probability to the point of asymptote to zero. Such a situation necessitates the assumption that $\lambda _{1}=\lambda _{2}=\dots =\lambda _{8} =0$. These constraints decrease our four operators (4.6) to

6.5 $$\begin{aligned} \left\{ \begin{aligned} P_{1}x&=\vartheta _{1}x, \\ P_{2}x&=\vartheta _{2}x, \\ P_{3}x&=\vartheta _{3}x, \\ P_{4}x&=\vartheta _{4}x, \\ P_{5}x&=\vartheta _{5}x, \\ P_{6}x&=\vartheta _{6}x \\ P_{7}x&=\vartheta _{7}x \\ P_{8}x&=\vartheta _{8}x \ \end{aligned}\right. \end{aligned}$$

By setting lambda values to zero in cases where habitual side preferences emerge, this condition prevents the model from being influenced by fixed behavioral reflexes. As a result, decision-making remains dynamically responsive to environmental cues rather than being constrained by preconditioned biases, thereby improving the model’s generalizability across different testing conditions. So, we can write (5.1) as

6.6 $$\begin{aligned} \mathcal {W}(x)= & \frac{1}{2}u_{1}x\mathcal {W}(\vartheta _{1}x) +\frac{1}{2}(1-u_{1})x\mathcal {W}(\vartheta _{2}x)\nonumber \\ & +\frac{1}{2}u_{2}x\mathcal {W}(\vartheta _{3}x) \nonumber \\ & +\frac{1}{2}(1-u_{2})x\mathcal {W}(\vartheta _{4}x) +\frac{1}{2}(1-x)u_{1} \mathcal {W}(\vartheta _{5}x)\nonumber \\ & +\frac{1}{2}(1-x)(1 - u_{1}) \mathcal {W}(\vartheta _{6}x), \nonumber \\ & +\frac{1}{2}(1-x)u_{2} \mathcal {W}(\vartheta _{7}x) +\frac{1}{2}(1-x)(1 - u_{2}) \mathcal {W}(\vartheta _{8}x), \end{aligned}$$

where $0<\vartheta _{1},\vartheta _{2},\vartheta _{3},\vartheta _{4},\vartheta _{5},\vartheta _{6}, \vartheta _{7}, \vartheta _{8}<1$, $u_{1},u_{2}\in \mathcal {A}$ and $\mathcal {W}:\mathcal {A}\rightarrow \mathbb {R}$ is an unknown function. The following are the Theorem 5.1’s corollaries.

#### Corollary 6.3

Let $0<\vartheta _{1},\vartheta _{2},\vartheta _{3},\vartheta _{4},\vartheta _{5},\vartheta _{6}, \vartheta _{7}, \vartheta _{8}<1$ and $u_{1},u_{2}\in \mathcal {A},$ with $\mathfrak {K}_{3}^{\star } <1$, where $\mathfrak {K}_{3}^{\star }$ is defined in (4.4). If an operator *Z* from $\Lambda$ associated for every $\mathcal {W}$ $\in$ $\Lambda$ and for all $x\in \mathcal {A}$ by

6.7 $$\begin{aligned} (Z\mathcal {W})(x)= & \frac{1}{2}u_{1}x\mathcal {W}(\vartheta _{1}x) +\frac{1}{2}(1-u_{1})x\mathcal {W}(\vartheta _{2}x)\nonumber \\ & +\frac{1}{2}u_{2}x\mathcal {W}(\vartheta _{3}x) \nonumber \\ & +\frac{1}{2}(1-u_{2})x\mathcal {W}(\vartheta _{4}x) +\frac{1}{2}(1-x)u_{1} \mathcal {W}(\vartheta _{5}x)\nonumber \\ & +\frac{1}{2}(1-x)(1 - u_{1}) \mathcal {W}(\vartheta _{6}x) \nonumber \\ & +\frac{1}{2}(1-x)u_{2}\mathcal {W}(\vartheta _{7}x) +\frac{1}{2}(1-x)(1 - u_{2}) \mathcal {W}(\vartheta _{8}x), \end{aligned}$$

and satisfies property ($\mathfrak {P}^{\star }$), then *Z* is a BCM.

#### Corollary 6.4

The stochastic Eq. (6.6) has a unique solution with $\mathfrak {K}_{3}^{\star } <1$, where $\mathfrak {K}_{3}^{\star }$ is given in (4.4), and there exists an operator *Z* defined in (6.7) satisfies property ($\mathfrak {P}^{\star }$). Furthermore, the iteration $\{\mathcal {W}_{n}\}$ ($\forall n\in \mathbb {N}$) in $\Lambda$, where $\mathcal {W}_{0}\in \Lambda$, is given by

6.8 $$\begin{aligned} (\mathcal {W}_{n})(x)= & \frac{1}{2}u_{1}x\mathcal {W}_{n-1}(\vartheta _{1}x) +\frac{1}{2}(1-u_{1})x\mathcal {W}_{n-1}(\vartheta _{2}x)\nonumber \\ & +\frac{1}{2}u_{2}x\mathcal {W}_{n-1}(\vartheta _{3}x) +\frac{1}{2}(1-u_{2})x\mathcal {W}_{n-1}(\vartheta _{4}x) \nonumber \\ & +\frac{1}{2}(1-x)u_{1} \mathcal {W}_{n-1}(\vartheta _{5}x) \nonumber \\ & +\frac{1}{2}(1-x)(1 - u_{1})\mathcal {W}_{n-1}(\vartheta _{6}x) \nonumber \\ & +\frac{1}{2}(1-x)u_{2}\mathcal {W}_{n-1}(\vartheta _{7}x) \nonumber \\ & +\frac{1}{2}(1-x)(1 - u_{2})\mathcal {W}_{n-1}(\vartheta _{8}x), \end{aligned}$$

converges to the unique solution of (6.6).

In the same way, if the rat frequently selects the food side, the chance of that event occurring increases. Therefore, we have

$$\begin{aligned} \lambda _{1} = \lambda _{2} = \dots = \lambda _{8} =1. \end{aligned}$$

In this situation, our four operators will be

6.9 $$\begin{aligned} \left\{ \begin{aligned} P_{1}x&=\vartheta _{1}x+(1-\vartheta _{1}), \\ P_{2}x&=\vartheta _{2}x+(1-\vartheta _{2}), \\ P_{3}x&=\vartheta _{3}x+(1-\vartheta _{3}), \\ P_{4}x&=\vartheta _{4}x+(1-\vartheta _{4}), \\ P_{5}x&=\vartheta _{5}x+(1-\vartheta _{5}), \\ P_{6}x&=\vartheta _{6}x+(1-\vartheta _{6}). \\ P_{7}x&=\vartheta _{7}x+(1-\vartheta _{7}), \\ P_{8}x&=\vartheta _{8}x+(1-\vartheta _{8}). \ \end{aligned}\right. \end{aligned}$$

Now, our functional Eq. (5.1) may be expressed as

6.10 $$\begin{aligned} \mathcal {W}(x)= & \frac{1}{2}u_{1}x\mathcal {W}(\vartheta _{1}x + (1-\vartheta _{1}))\nonumber \\ & +\frac{1}{2}(1-u_{1})x\mathcal {W}(\vartheta _{2}x + (1-\vartheta _{2})) \nonumber \\ & + \frac{1}{2}u_{2}x\mathcal {W}(\vartheta _{3}x + (1-\vartheta _{3}))\nonumber \\ & + \frac{1}{2}(1-u_{2})x\mathcal {W}(\vartheta _{4}x + (1-\vartheta _{4})) \nonumber \\ & + \frac{1}{2}(1-x)u_{1} \mathcal {W}(\vartheta _{5}x + (1-\vartheta _{5}))\nonumber \\ & +\frac{1}{2}(1-x)(1 - u_{1}) \mathcal {W}(\vartheta _{6}x + (1-\vartheta _{6})),\nonumber \\ & + \frac{1}{2}(1-x)u_{2} \mathcal {W}(\vartheta _{7}x + (1-\vartheta _{7}))\nonumber \\ & +\frac{1}{2}(1-x)(1 - u_{2})\mathcal {W}(\vartheta _{8}x + (1-\vartheta _{8})), \end{aligned}$$

where $0<\vartheta _{1}, \vartheta _{2}, \vartheta _{3}, \vartheta _{4},\vartheta _{5}, \vartheta _{6}, \vartheta _{7}, \vartheta _{8} <1$, $u_{1}, u_{2} \in \mathcal {A}$ and $\mathcal {W}:\mathcal {A}\rightarrow \mathbb {R}$ is an unknown function. The following outcomes are the findings of Theorem 5.1.

#### Corollary 6.5

Let $0<\vartheta _{1}, \vartheta _{2}, \vartheta _{3}, \vartheta _{4}, \vartheta _{5}, \vartheta _{6}, \vartheta _{7}, \vartheta _{8}<1$ and $u_{1}, u_{2} \in \mathcal {A}$ with $\mathfrak {K}_{4}^{\star } <1$, where $\mathfrak {K}_{4}^{\star }$ is defined in (4.5). If an operator *Z* from $\Lambda$ associated for every $\mathcal {W}$ $\in$ $\Lambda$ and for all $x\in \mathcal {A}$ by

6.11 $$\begin{aligned} (Z\mathcal {W})(x)= & \frac{1}{2}u_{1}x\mathcal {W}(\vartheta _{1}x +(1-\vartheta _{1}))\nonumber \\ & +\frac{1}{2}(1-u_{1})x\mathcal {W}(\vartheta _{2}x +(1-\vartheta _{2})) \nonumber \\ & + \frac{1}{2}u_{2}x\mathcal {W}(\vartheta _{3}x + (1-\vartheta _{3}))\nonumber \\ & + \frac{1}{2}(1-u_{2})x\mathcal {W}(\vartheta _{4}x + (1-\vartheta _{4})) \nonumber \\ & +\frac{1}{2}(1-x)u_{1} \mathcal {W}(\vartheta _{5}x + (1-\vartheta _{5}))\nonumber \\ & +\frac{1}{2}(1-x)(1 - u_{1}) \mathcal {W}(\vartheta _{6}x + (1-\vartheta _{6})),\nonumber \\ & +\frac{1}{2}(1-x)u_{2} \mathcal {W}(\vartheta _{7}x + (1-\vartheta _{7}))\nonumber \\ & +\frac{1}{2}(1-x)(1 - u_{2}) \mathcal {W}(\vartheta _{8}x + (1-\vartheta _{8})), \end{aligned}$$

and satisfies property ($\mathfrak {P}^{\star }$), then *Z* is a BCM.

#### Corollary 6.6

The Eq. (6.10) has a unique solution claiming that $\mathfrak {K}_{4}^{\star } <1$, where $\mathfrak {K}_{4}^{\star }$ is defined in (4.5), and there exists an operator *Z* defined in (6.11) satisfies property ($\mathfrak {P}^{\star }$). Furthermore, the iteration $\{\mathcal {W}_{n}\}$ ($\forall n\in \mathbb {N}$) in $\Lambda$, where $\mathcal {W}_{0}\in \Lambda$, is given by

6.12 $$\begin{aligned} (\mathcal {W}_{n})(x)= & \frac{1}{2}u_{1}x\mathcal {W}_{n-1}(\vartheta _{1}x+(1-\vartheta _{1}))\nonumber \\ & +\frac{1}{2}(1-u_{1})x\mathcal {W}_{n-1}(\vartheta _{2}x+(1-\vartheta _{2}))\nonumber \\ & +\frac{1}{2}u_{2}x\mathcal {W}_{n-1}(\vartheta _{3}x+(1-\vartheta _{3}))\nonumber \\ & +\frac{1}{2}(1-u_{2})x\mathcal {W}_{n-1}(\vartheta _{4}x+(1-\vartheta _{4})) \nonumber \\ & +\frac{1}{2}(1-x)u_{1} \mathcal {W}_{n-1}(\vartheta _{5}x+(1-\vartheta _{5}))\nonumber \\ & +\frac{1}{2}(1-x)(1 - u_{1}) \mathcal {W}_{n-1}(\vartheta _{6}x+(1-\vartheta _{6})), \nonumber \\ & +\frac{1}{2}(1-x)u_{2} \mathcal {W}_{n-1}(\vartheta _{7}x+(1-\vartheta _{7}))\nonumber \\ & +\frac{1}{2}(1-x)(1 - u_{2}) \mathcal {W}_{n-1}(\vartheta _{8}x+(1-\vartheta _{8})), \end{aligned}$$

converges to the unique solution of (6.10).

## Performance validation with deep learning methods

To analyze and classify rat movements (left or right), we applied two widely recognized deep learning approaches: Convolutional Neural Networks-Long Short-Term Memory (CNN-LSTM) and Convolutional Neural Networks-Gated Recurrent Unit (CNN-GRU) (see Wu et al. 2021; Ullah et al. 2024 for more detail).

### Data collection and processing

The dataset initially consisted of video recordings capturing rat movements toward rewards in a controlled environment. We developed a method to extract frames from the videos, resulting in 153 images of left movements and 105 images of right movements. However, this dataset was insufficient for training deep learning models, which require a larger volume of data to generalize effectively during training.

To overcome this limitation, we applied data augmentation techniques to generate additional images while preserving the characteristics of the original dataset. This method enhanced the diversity of the training set, improving model generalization. The augmentation techniques included rotation, flipping, zooming, cropping, brightness and contrast adjustment, and noise injection:

- **Rotation:** Rotating images to improve the model’s resilience to varying perspectives of movement.
- **Flip:** Flipping images vertically or horizontally to make the model more robust against different movement orientations.
- **Zoom:** Cropping and resizing images to simulate zooming, aiding the model in tracking movement.
- **Crop:** Randomly cropping portions of the images to help the model focus on different areas of the objects.
- **Brightness and Contrast Adjustment:** Modifying brightness and contrast to simulate different lighting conditions.
- **Noise Injection:** Adding random noise to make the model more resistant to variations in input data.

### Statistical analysis

To address the high dimensionality of the dataset, a strategic approach was adopted to ensure the analysis remained concise and focused. Including all features (Feature_0, Feature_1, Feature_2, $\dots$, Feature_127), which are extracted from the images, would have resulted in an overly complex and lengthy report. Instead, the top five features exhibiting the highest variability-Feature_0, Feature_8, Feature_16, Feature_96, and Feature_104-were selected for detailed examination based on their standard deviation. A t-test was employed to compare these features between the left and right turn datasets. The analysis revealed that Feature_8 and Feature_16 demonstrated statistically significant differences $(p < 0.001)$, highlighting their potential as robust discriminators between the two movement types.

To further validate these findings, the Kolmogorov-Smirnov (KS) test was applied to compare the cumulative distributions of the left and right turn movements across the top five features. The results confirmed that Feature_0, Feature_8, and Feature_16 exhibited the highest KS statistics and the lowest p-values $(p < 0.001)$, indicating substantial differences in their distributions between the two movement types. This further underscores their discriminative potential for classification tasks. Table 4 represents a summary of the key statistical outcomes for these features.

**Table 4:** Statistical comparison of top five features between left and right turns

Feature	Mean	Mean	SD	SD	t-statistic	p-value	KS-statistic
	(Right)	(Left)	(Right)	(Left)			
Feature_0	55.523	48.662	59.760	55.626	2.657	$7.94 \times 10^{-3}$	0.182
Feature_8	96.290	84.622	59.836	64.899	4.180	$3.04 \times 10^{-5}$	0.145
Feature_16	102.822	81.420	61.131	60.996	7.837	$7.43 \times 10^{-15}$	0.184
Feature_96	61.703	60.367	60.117	59.277	0.500	$6.17 \times 10^{-1}$	0.110
Feature_104	105.254	100.396	61.382	59.588	1.796	$7.27 \times 10^{-2}$	0.145

**Fig. 10:**
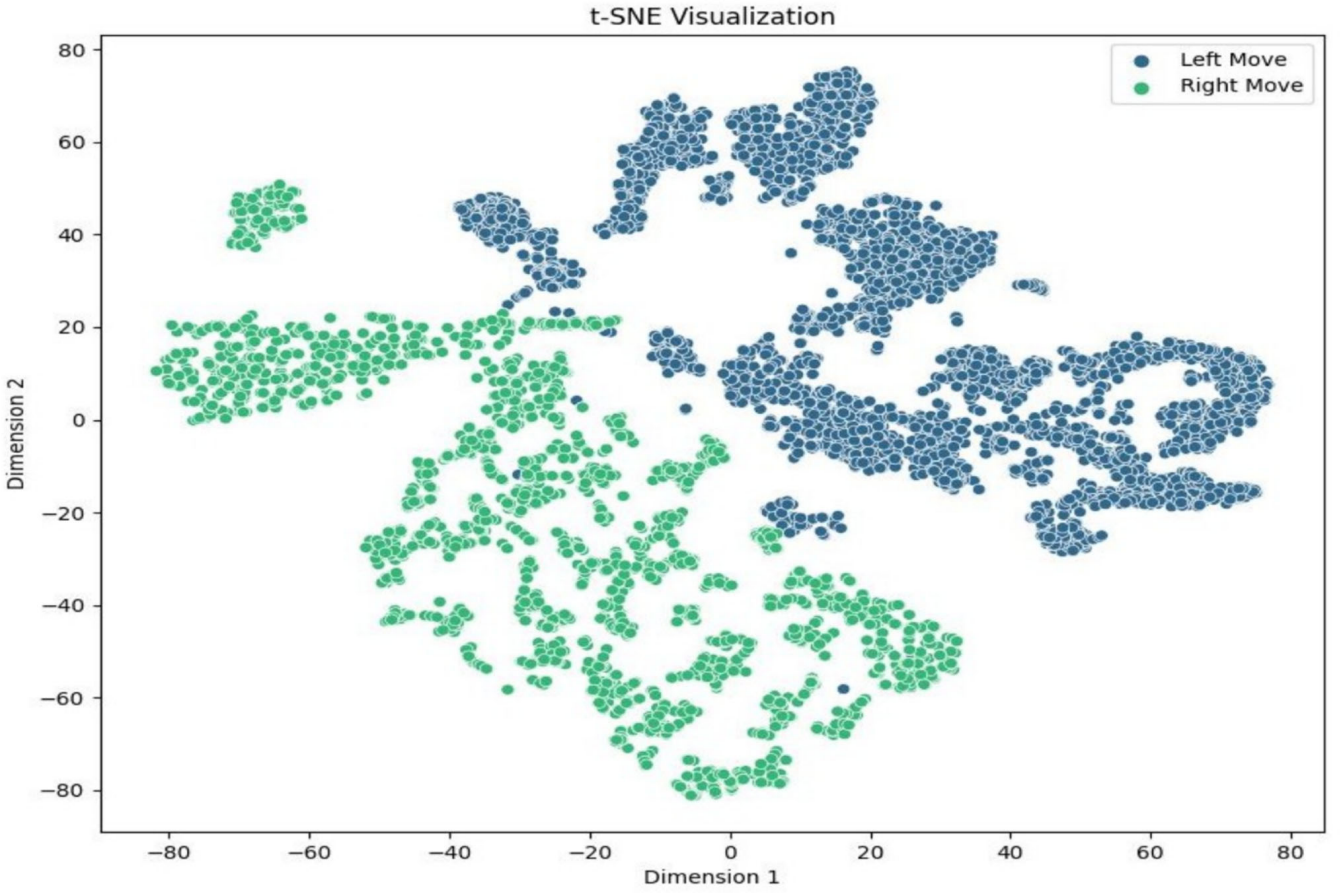
The dataset is shown using t-SNE with perplexity $p = 30$. The t-SNE approach transforms high-dimensional data into lower-dimensional space, exposing cluster structures and interactions between data points. This visualization aids in determining class separability and the efficacy of feature representation

**Fig. 11:**
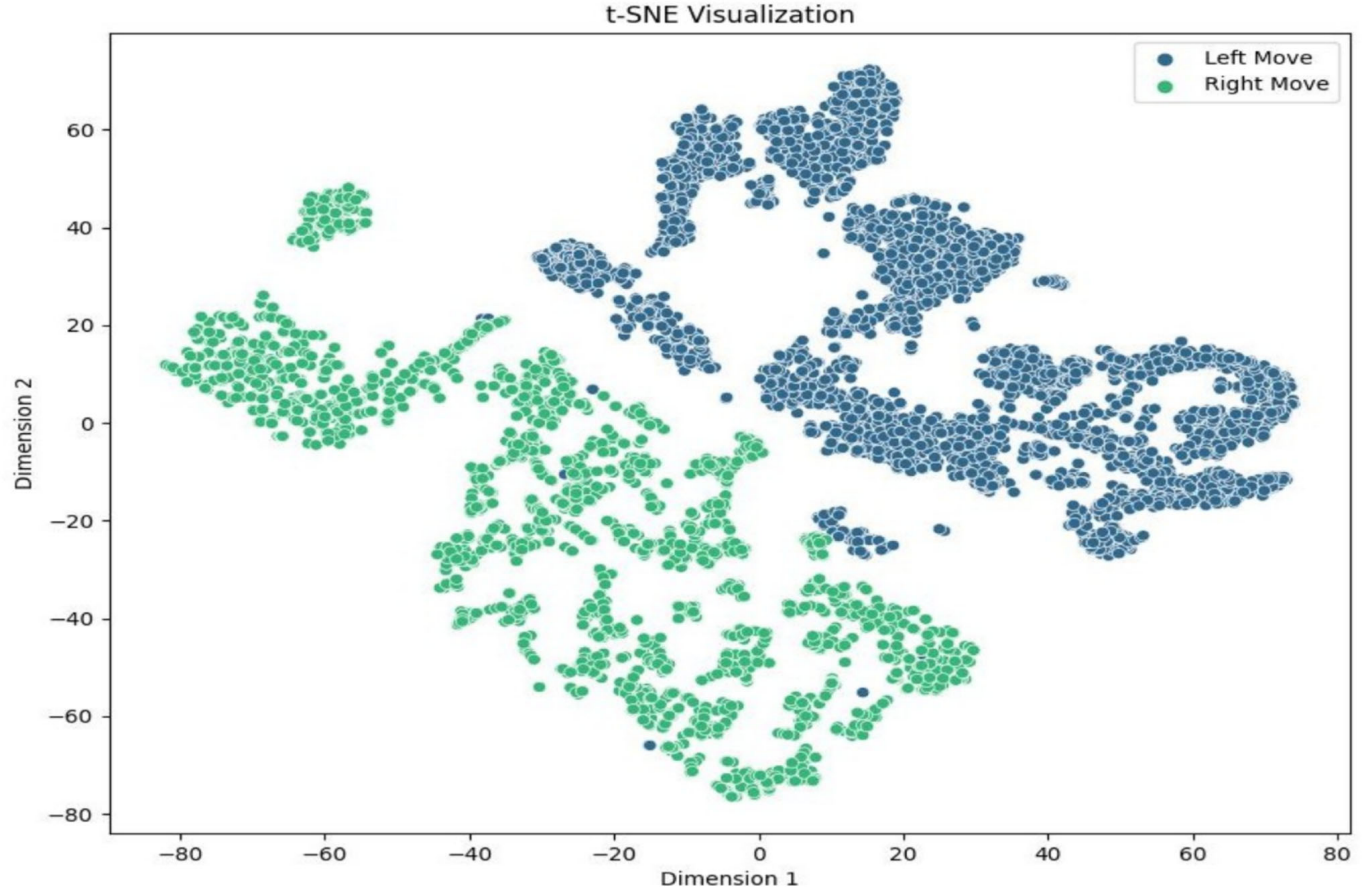
The dataset is shown using t-SNE with perplexity $p = 40$. The t-SNE approach transforms high-dimensional data into lower-dimensional space, exposing cluster structures and interactions between data points

**Fig. 12:**
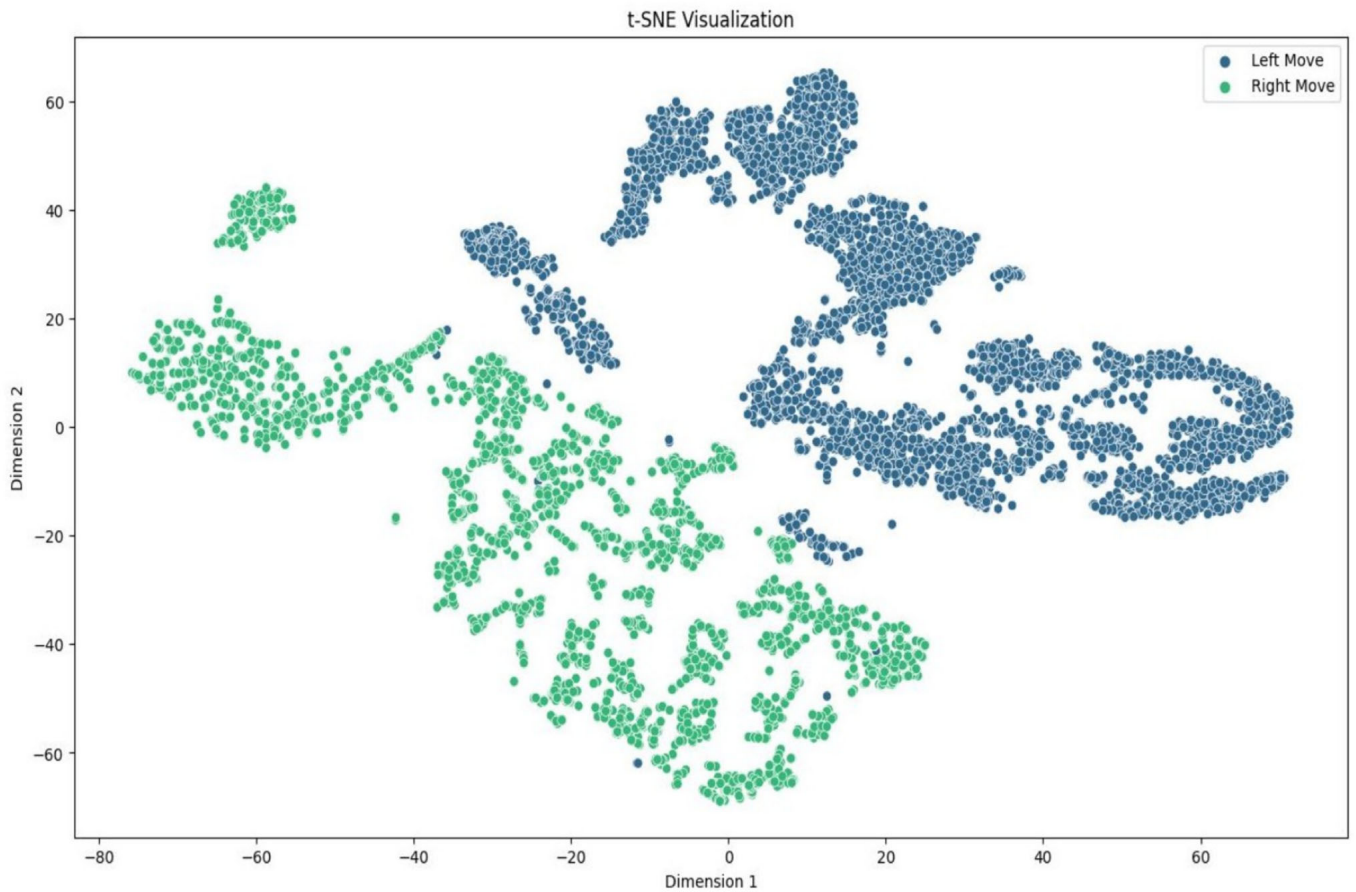
The dataset is shown using t-SNE with perplexity $p = 50$. The t-SNE approach transforms high-dimensional data into lower-dimensional space, exposing cluster structures and interactions between data points

To explore the separability of the datasets further, t-Distributed Stochastic Neighbor Embedding (t-SNE) was employed to visualize high-dimensional data in lower-dimensional space. Figures 10, 11 and 12 illustrate the clustering of left and right movements under different perplexity values $(p=30, 40, 50)$. The visual separation between the movements indicates the extracted features’ effectiveness in distinguishing the two types. The distinct boundaries of the clusters demonstrate the robustness of the feature extraction process, confirming the validity of the machine learning models used.

**Fig. 13:**
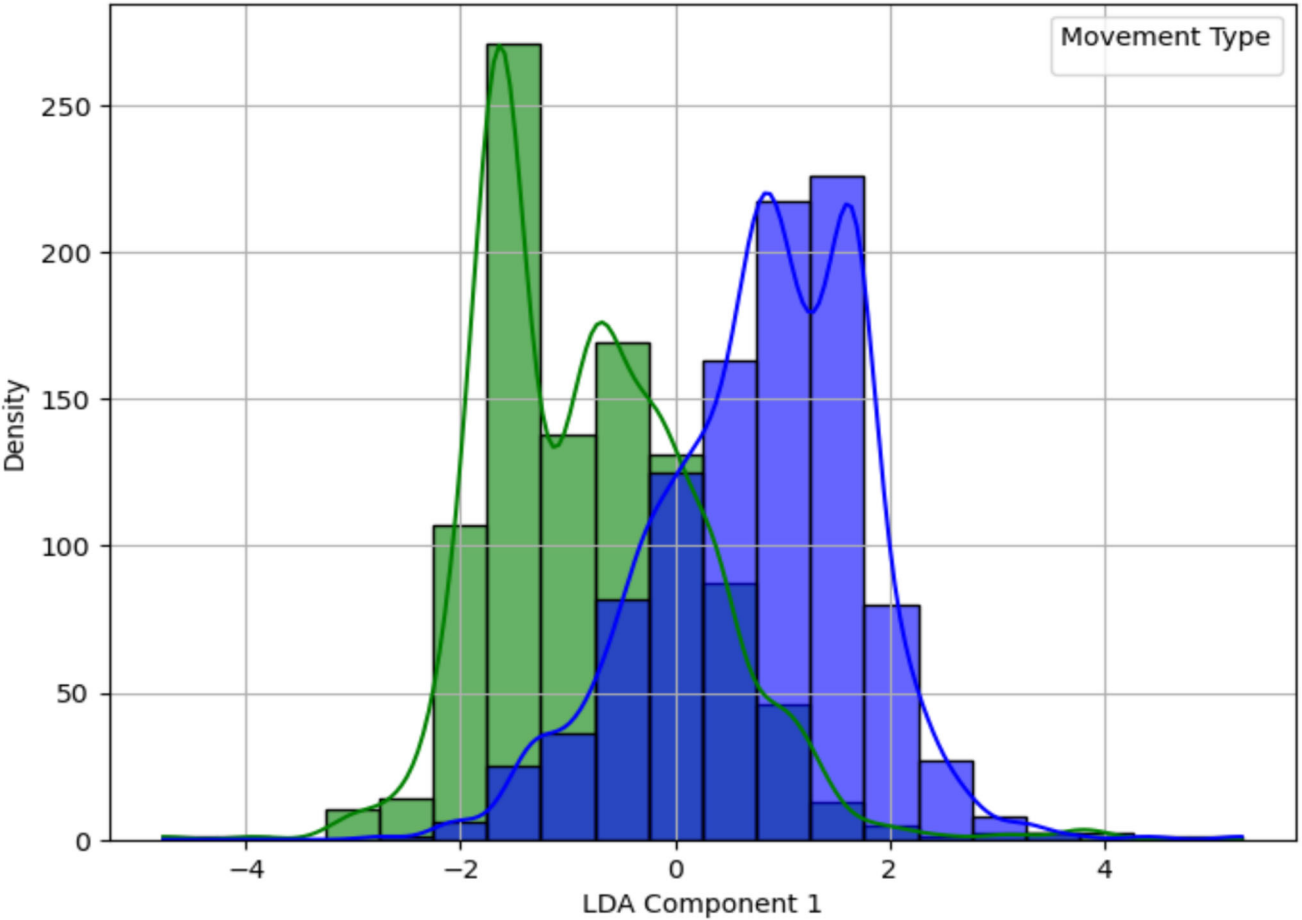
LDA projection of left and right turn features

In addition, Linear Discriminant Analysis (LDA) was performed to project the data onto a single discriminative axis. Figure 13 illustrates the distribution of the left and right turn datasets with distinct color coding: light/dark blue represents the left turn movement, while green represents the right turn movement. The overlapping regions in the plot indicate areas where the two classes are less separable using a linear boundary, while the distinct peaks highlight regions with clearer differentiation. Unlike PCA, which maximizes variance, LDA focuses on maximizing class separation by identifying an optimal linear boundary. The LDA plot demonstrated superior separation compared to the PCA projection, underscoring the contribution of specific features to class differentiation.

**Fig. 14:**
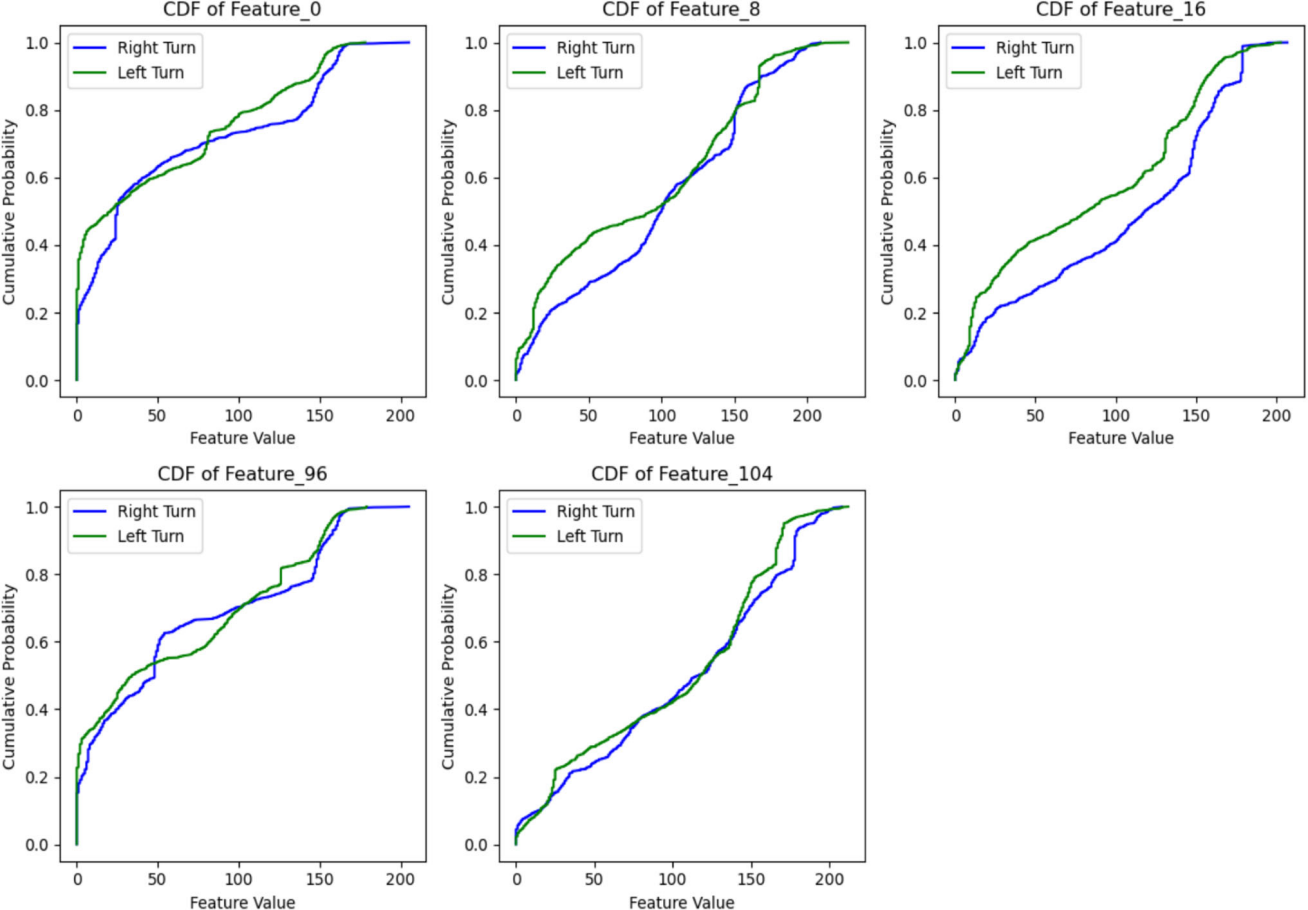
KS test: CDF comparison of left and right turn features

The Cumulative Distribution Function (CDF) plots illustrate that greater separation between the curves corresponds to stronger feature discriminability (see Fig. 14). While Feature_96 and Feature_104 also showed some separation, their differences were less pronounced compared to Feature_0, Feature_8, and Feature_16. These findings emphasize the importance of feature selection in movement classification and suggest that leveraging these key features can significantly enhance the predictive accuracy of machine learning models.

### Results analysis

**Fig. 15:**
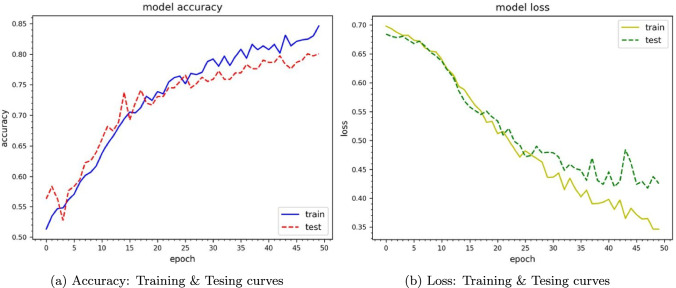
Training and testing performance curves for the CNN-LSTM model. **a** Accuracy curves show the variation in training and testing accuracy over epochs, demonstrating the model’s learning progress and convergence. **b** The training and testing loss changes over epochs are represented by loss curves, which illustrate how well the model reduces errors and generalizes to unseen data

The augmented dataset was processed using the designed deep learning models, CNN-LSTM and CNN-GRU. Figure 15 presents the epoch curves for training and testing data. In Part (a), training accuracy is depicted in blue, while testing accuracy is shown in red. These graphs illustrate the model’s performance over time. Initially, the training curve starts at 15%, and the testing curve at 63%. The testing accuracy dips to 40% before gradually increasing with each epoch. The training curve follows a steady progression, while the testing curve fluctuates, dipping to 70% at epoch 20 before rising again. After a few more epochs, both curves stabilize around 84%, indicating consistent model performance.

Part (b) shows the training and testing losses for each epoch. Training loss is shown in yellow, and testing loss is shown in green. Both curves begin at around 70% and steadily decrease over the epochs. Although the testing curve briefly rises to 55% by the 35th epoch, it quickly decreases. Ultimately, both curves converge to a loss of around 19%. These loss curves complement the accuracy curves, confirming that the proposed approach achieves strong performance with minimal loss.

**Fig. 16:**
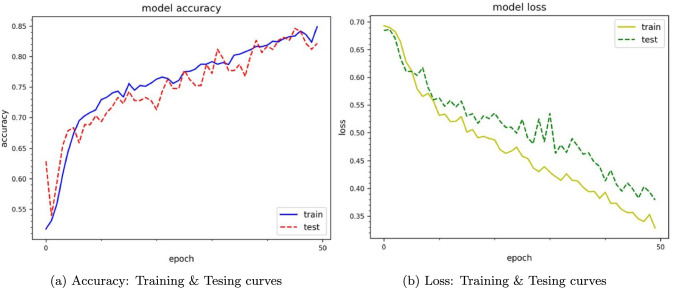
Training and testing performance curves for the CNN-GRU model. **a** Accuracy curves show the variation in training and testing accuracy over epochs, demonstrating the model’s learning progress and convergence. **b** The training and testing loss changes over epochs are represented by loss curves, which illustrate how well the model reduces errors and generalizes to unseen data

Similarly, Fig. 16 displays the training and testing curves for the CNN-GRU model. The training curve follows a regular, increasing pattern in line with the number of epochs. The testing curve, while occasionally showing rapid fluctuations, stabilizes and mirrors the behavior of the training curve. Overall, both curves show performance between 50% and 82%. The loss curves demonstrate a reduction from 70% to 20%, further supporting the model’s effectiveness.

Five metrics were used to evaluate model performance: precision, recall, F1-score, accuracy, and confusion matrices. True Positives (TP) and True Negatives (TN) represent correct classifications for left and right movements, while False Positives (FP) and False Negatives (FN) represent misclassifications. Accuracy was calculated by dividing correctly classified instances by the total number of instances, with the formulas provided in Eqs. (7.1)–(7.4).

7.1 $$\begin{aligned} Recall= & \frac{FP}{(FP+TN)} \end{aligned}$$

7.2 $$\begin{aligned} Precision= & \frac{TP}{(TP+FP)} \end{aligned}$$

7.3 $$\begin{aligned} F1-score= & \frac{(2*TP)}{(2TP+FP+FN)} \end{aligned}$$

7.4 $$\begin{aligned} Accuracy= & \frac{(TP+TN)}{(TP+TN+FP+FN)} \end{aligned}$$

Table 5 displays the performance metrics for the CNN-LSTM model, where precision, recall, and F1-score for left movement are 81.18%, 81.51%, and 82.24%, respectively. For right movement, the corresponding values are 83.12%, 83.14%, and 81.76%, with an overall accuracy of 82.24%. Table 6 shows the CNN-GRU model performance, with left movement values of 81.23%, 80.82%, and 80.44%, and right movement values of 77.28%, 77.12%, and 80.61%, yielding an overall accuracy of 80.52%. These results demonstrate the proposed method’s capability to classify rat movements accurately.

**Fig. 17:**
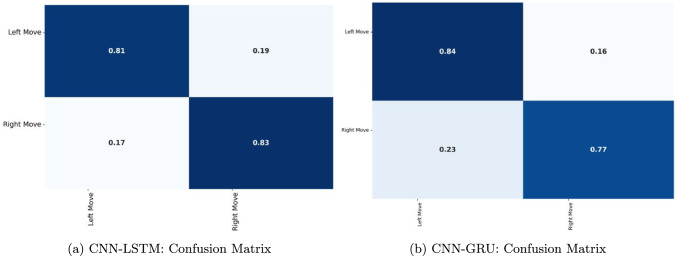
Confusion matrices for the classification performance of the CNN-LSTM and CNN-GRU models. **a** CNN-LSTM: The confusion matrix depicts the CNN-LSTM model’s performance by showing the amount of true positives, false positives, true negatives, and false negative predictions in each class. **b** CNN-GRU: The confusion matrix for the CNN-GRU model, similarly illustrating the model’s classification performance with the respective counts of true and false predictions. These matrices present a comprehensive understanding of the models’ accuracy, class balance, and potential misclassifications

Confusion matrices provide further insight into classification performance, detailing TP, TN, FP, and FN rates. Figure 17 shows that the CNN-LSTM model correctly classifies 81% of left movements and 83% of right movements, with respectively 19% and 17% misclassification rates. For CNN-GRU, left movements are correctly classified 84% of the time, with an error rate of 16%, while right movements show a 77% accuracy and a 23% error rate. These results confirm the models’ robustness in tracking and classifying rat movements.

**Table 5:** Performance measures using CNN-LSTM

Movements	Precision	Recall	F1-measure	Accuracy%
Left move	0.8118	0.8151	0.8224	0.8224
Right move	0.8312	0.8314	0.8176

**Table 6:** Performance measures using CNN-GRU

Movements	Precision	Recall	F-measure	Accuracy%
Left move	0.8123	0.8082	0.8044	0.8052
Right move	0.8061	0.7728	0.7712	

Table 7 shows the evaluation metrics for a model that employs a transformer encoder to categorize Left Move and Right Move. The model obtained a Left Move precision score of 82.07% and a Right Move precision score of 82.14%. These results are quite similar, indicating that the model is equally effective at accurately projecting positive events for both motions. The model showed a higher recall for Left Move (84.05%) than for Right Move (80.31%). This demonstrates that the model identified more positive instances of Left Move than Right Move. The F1-measure for Left Move was 82.05%, while Right Move had an F1 of 81.36%. This measure combines precision and recall. Although the Left Move F1-measure is slightly lower, both values are close, demonstrating that the model’s overall performance remains stable over the two movements. Finally, the model achieved an Accuracy of 82.15%, which means that around 82.15% of all predictions provided by the model throughout both movements are correct.

A comparison of the Transformer Encoder and CNN-LSTM models reveals that each model has distinct advantages in certain areas. The Transformer Encoder has a high left move recall rate (84.05%) and a slightly higher precision rate (82.07%). While both models achieve better F1-measures, the CNN-LSTM model has an outperformance in Precision for Right Move (83.12%). The Transformer Encoder consistently obtains an accuracy of 82.15%, whereas the CNN-LSTM model is slightly more accurate for Left Move (82.24%) but less accurate for Right Move (81.76%). In general, both models exhibit exceptional performance, with each model excelling in distinct areas based on the movement style.

We used cross-validation (KFold with 5 splits) to assess the models’ generalization performance, ensuring that they did not overfit the training data. Cross-validation evaluates the performance of models using previously unknown data by dividing the dataset into multiple training and testing segments. The experiment tests for overfitting by comparing training and test accuracy. If training accuracy is significantly higher than test accuracy, the model could be overfitting. This comparison demonstrates that the models can accurately generalize to new data. Table 8 compares the performance of two machine learning models, Decision Tree (DT) and Support Vector Machine (SVM), in detecting mouse movements. The Decision Tree model outperforms the Right Move in terms of recall and precision, yielding a total accuracy of 81.39%. The Decision Tree obtains an accuracy of 80.83%, whereas the SVM model has slightly lower recall and precision scores for both motions. The F1-measure is quite evenly distributed between the two models, implying that they have similar classification efficiency.

The presented experiments demonstrate the CNN-LSTM model’s contribution when compared to other models such as the Transformer Encoder, Decision Tree, and SVM. The CNN-LSTM model is particularly effective in encoding spatial and temporal dependencies in the data, which enhances its performance, particularly in terms of precision for Right Move, by combining convolutional and LSTM layers. The CNN-LSTM outperforms the Transformer Encoder in precision for Right Move, despite its strong recall performance, particularly for Left. Deep learning models outperform DT and SVM because they are better able to detect sequential patterns, which leads to more accurate classifications.

**Table 7:** Performance measures using transformer encoder

Movements	Precision	Recall	F1-measure	Accuracy%
Left move	0.8207	0.8405	0.8205	0.8215
Right move	0.8214	0.8031	0.81364

**Table 8:** Performance measures using decision tree and SVM

Movements	Precision	Recall	F1-measure	Accuracy %
**Decision tree**				
Left move	0.8065	0.8197	0.8130	0.8139
Right move	0.8103	0.7966	0.8034	
**SVM**				
Left move	0.7848	0.7770	0.7809	0.8083
Right move	0.7718	0.7797	0.7757	

## Conclusion and open problems

This study proposed a cognitive framework for modeling rat decision-making behavior in $\mathbb {T}$-mazes by combining stochastic processes with deep learning methods. The model builds on Wyckoff’s stochastic formulation to represent probabilistic response shifts across trials under reinforcement contingencies. The existence and uniqueness of solutions were established through the Banach fixed-point theorem, ensuring the mathematical consistency of the system. A comparative analysis between Picard iteration and Monte Carlo simulations demonstrated close agreement, supporting the numerical stability of the model.

We employed deep neural architectures, such as CNN-LSTM and CNN-GRU models, which were trained on trajectory sequences derived from experimental recordings to examine behavioral data. These models achieved notable classification performance, outperforming standard approaches. Statistical preprocessing and nonlinear dimensionality reduction using t-SNE facilitated the analysis of feature distributions across behavioral states, offering interpretable structure within high-dimensional data.

The results suggest that integrating stochastic modeling with data-driven neural methods can effectively capture the probabilistic structure and temporal dynamics of navigational behavior. This approach enables the analysis of individual learning patterns without relying on strong parametric assumptions or oversimplified behavioral rules.

Several open problems remain for further investigation.

1. How does the decision-making process evolve in trial *k* if the rat does not move toward the left or right compartment?
2. A fundamental aspect of functional equations is their stability, particularly within the Ulam-Hyers and Ulam-Hyers-Rassias frameworks (see Brzdek 2023; Choubin and Javanshiri 2021). The stability properties of the following equation remain unresolved and warrant further analysis.
  $$\begin{aligned} \mathcal {W}(x)= & \frac{1}{2}u_{1}x\mathcal {W}(\vartheta _{1}x+(1-\vartheta _{1})\lambda _{1})\\ & +\frac{1}{2}(1-u_{1})x\mathcal {W}(\vartheta _{2}x+(1-\vartheta _{2})\lambda _{2}) \\ & +\frac{1}{2}u_{2}x\mathcal {W}(\vartheta _{3}x+(1-\vartheta _{3})\lambda _{3})\\ & +\frac{1}{2}(1-u_{2})x\mathcal {W}(\vartheta _{4}x+(1-\vartheta _{4})\lambda _{4}) \\ & +\frac{1}{2}(1-x)u_{1}\mathcal {W}(\vartheta _{5}x+(1-\vartheta _{5})\lambda _{5})\\ & + \frac{1}{2}(1-x)(1-u_{1}) \mathcal {W}(\vartheta _{6}x+(1-\vartheta _{6})\lambda _{6})\nonumber \\ & + \frac{1}{2}(1-x)u_{2} \mathcal {W}(\vartheta _{7}x+(1-\vartheta _{7})\lambda _{7})\\ & + \frac{1}{2}(1-x)(1-u_{2}) \mathcal {W}(\vartheta _{8}x+(1-\vartheta _{8})\lambda _{8}), \end{aligned}$$
  where $$\mathcal {W}:\mathcal {A}\rightarrow \mathbb {R}$$, $$0<\vartheta _{1},\vartheta _{2},\vartheta _{3},\vartheta _{4},\vartheta _{5},\vartheta _{6},\vartheta _{7},\vartheta _{8}<1$$ and $$\lambda _{k}\ (k=1,2,...,8),u_{1},u_{2}\in \mathcal {A}$$.

## Data Availability

The data that support the findings of this study can be accessed from here: https://github.com/Farhankhancs/AliTurab.
